# Super-resolution imaging of limited-size objects

**DOI:** 10.1038/s41566-025-01839-2

**Published:** 2026-02-16

**Authors:** Taeyong Chang, Giorgio Adamo, Nikolay I. Zheludev

**Affiliations:** 1 Centre for Disruptive Photonic Technologies, SPMS and TPI, Nanyang Technological University, Singapore, Singapore; 2 Optoelectronics Research Centre and Centre for Photonic Metamaterials, University of Southampton, Southampton, UK; 3 Hagler Institute for Advanced Study, Texas A&M University, College Station, TX USA

**Keywords:** Super-resolution microscopy, Sub-wavelength optics, Imaging and sensing

## Abstract

Label-free, far-field super-resolution imaging can be achieved by exploiting prior knowledge about an object, such as sparsity, or by using information accumulated from similar object classes. Here we show that simply knowing that an object is confined within a limited spatial extent is sufficient to surpass the Abbe–Rayleigh diffraction limit: for a fixed photon budget, smaller objects can be resolved with higher spatial resolution. To demonstrate this, we develop a limited-size object microscopy (LSOM) technique. The method relies on representing the coherently scattered field from the object within a limited field of view with Slepian–Pollak functions, a family of prolate spheroidal wavefunctions. The coefficients of such functions can then be recovered from diffraction-limited measurements. We experimentally demonstrate down to *λ*/8 resolution (where *λ* is the wavelength) for subwavelength structures and analyse the performance limits of the method using information theory. The technique requires no assumptions about the object’s shape or complexity and does not rely on labels, making it broadly applicable to the study of isolated nano-objects.

## Abstract (web summary)

Limited-size object microscopy (LSOM) enables label-free super-resolution imaging of isolated nano-objects with a resolution as low as *λ*/8 under the sole assumption of the limited size of the imaged object.

## Main

The widely used resolution limit of approximately half the wavelength (*λ*/2), introduced more than a century ago by Abbe^1^, Helmholtz^2^ and Rayleigh^3^, remains a standard benchmark in optical imaging. Its endurance as a resolution criterion is not due to being a fundamental physical bound but rather to its practical relevance for conventional microscopy^4,5^. Overcoming this limit without resorting to invasive or sample-altering techniques—such as near-field scanning or fluorescent labelling—remains a central challenge.

Information theory provides a quantitative framework for analysing the possibility of achieving super-resolution in label-free, far-field imaging, by treating the imaging system as an information channel^6–8^. Within this framework, super-resolution becomes attainable if sufficient prior information about the object is available and the measurement accuracy is high enough^6–12^. In particular, the impact of a finite object size on achievable resolution has been investigated theoretically since the 1950s^13–28^, predicting that arbitrarily high resolution is, in principle, possible. One route to achieving this is to represent a spatially confined object using Slepian–Pollak functions (prolate spheroidal wavefunctions)^29,30^, whose coefficients can—in principle—be uniquely recovered from diffraction-limited measurements^17–19^.

However, all theoretical studies have consistently emphasized that the required measurement accuracy is extremely demanding and that achieving meaningful super-resolution may not be feasible in practice^9–12^. As a consequence, and to the best of our knowledge, no experimental demonstrations of label-free deep super-resolution imaging, without structured illumination, where the sole assumption is the limited spatial extent of the object, have yet been achieved.

Here we report the experimental realization of label-free, far-field optical imaging with resolutions of *λ*/7 and *λ*/8 for two-dimensional (2D) and one-dimensional (1D) objects, respectively, under the sole assumption that the object is confined within a sub-wavelength region, smaller than 0.8*λ* for the proof-of-principle demonstrations. To achieve this, we use a reconfigurable mask to separate the Slepian–Pollak components of coherent light (*λ* = 638 nm) scattered by the object, measure them individually and correct for optical distortions in the measurement apparatus. Because our method can be understood as the reverse process of superoscillatory hotspot generation, we explicitly compare it with super-resolution microscopy based on structured superoscillatory illumination^31–35^. Furthermore, by recasting the proposed limited-size object microscopy (LSOM) process in an information-theoretic framework, we derive a fundamental performance bound, identify the trade-off between resolution, photon budget and object size limit, show that smaller objects can be imaged at higher resolution for a fixed photon budget and establish the experimental requirements for achieving super-resolution.

## Results

### Analogy between superoscillatory hotspot generation and LSOM

**Fig. 1:**
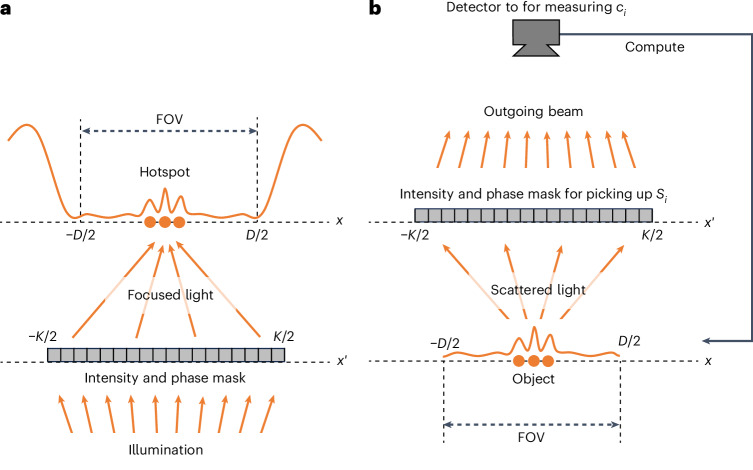
Concept of LSOM as the reverse process of superoscillatory hotspot generation. **a**, Superoscillatory hotspot generation. A tailored source profile is created in the Fourier plane *x*′, within the limited band *K*, by a magnitude and phase mask and is used to generate an arbitrary wave profile that contains deeply subwavelength features within the FOV of size *D* on the object plane *x*. **b**, LSOM. An arbitrary source profile within *D* at the object plane *x*, arising from light scattered by an object and containing deeply subwavelength features, generates a unique wave profile in the Fourier plane *x*′, within the band *K*. By measuring the Slepian–Pollak coefficients of the field amplitude in the Fourier plane, the source profile at the object plane can be estimated. In **a** and **b**, the source–wave relations are reciprocal configurations.

The LSOM process demonstrated here can be understood as a reverse process of superoscillatory hotspot generation (Fig. 1). It is known that an arbitrary wave profile—including superoscillatory patterns with arbitrarily small hotspots—can be generated within a field of view (FOV) of size *D* in the object plane (coordinate *x*) by engineering an intensity and phase mask within a band-limited region *K* on the Fourier plane (coordinate *x*′), as sketched in Fig. 1a (refs. ^36,37^). Reciprocally, in LSOM (Fig. 1b), light scattered from an object confined within the FOV of size *D* in the plane (*x*) can be converted into a propagating plane wave by applying an appropriate magnitude and phase correction with the same band-limited region *K* at the Fourier plane (*x*′) (Supplementary Text 1).

Each Slepian–Pollak function (*S*_*i*_) is an eigenfunction of the finite Fourier transformation operator, ${\boldsymbol{ {\mathcal F} }}$, changing only by a scalar factor *γ*_*i*_ under propagation, ${\boldsymbol{ {\mathcal F} }}[{S}_{i}]={\gamma }_{i}{S}_{i}$ (refs. ^29,30^). Wave propagation from the object to the Fourier plane in a diffraction-limited optical system is equivalent to applying this finite Fourier transform.

In the superoscillatory hotspot case, one can synthesize a desired field profile ${\sum }_{i}{c}_{i}{S}_{i}(x)$ with arbitrarily small hotspots FOV of size *D* by preparing a mask profile ${\sum }_{i}{c}_{i}{\gamma }_{i}^{-1}{S}_{i}(x{\prime} )$ that superposes Slepian–Pollak modes with weights *γ*_*i*_ (ref. ^36^). Non-zero amplitudes are allowed outside *D*. These hotspots have been exploited for superoscillatory super-resolution microscopy^31–35^.

In the case of LSOM, the optical near-field profile of an unknown confined object can be approximated by a finite Slepian–Pollak series ${\sum }_{i}{c}_{{\rm{e}},i}{S}_{i}(x)={\sum }_{i}{c}_{{\rm{m}},i}{\gamma }_{i}^{-1}{S}_{i}(x)$, by measuring the Slepian–Pollak coefficients of the wave, *c*_m,*i*_, through a mask (Supplementary Text 1)^17–19^. In practice, the coefficients *c*_e,*i*_ are an estimate of the true coefficients *c*_o,*i*_ derived from the measured coefficients *c*_m*,i*_, which inherently include a measurement uncertainty *ε*_*i*_, for example, *c*_e,*i*_ = *c*_m*,i*_/*γ*_*i*_ = (*γ*_*i*_*c*_o,*i*_ + *ε*_*i*_)/*γ*_*i*_. Because |*γ*_*i*_| decreases rapidly with the index *i* (refs. ^11,12,22^), it becomes difficult to recover accurately high-order coefficients: even small measurement uncertainties *ε*_*i*_ can be strongly amplified through division by *γ*_*i*_.

### Fundamental performance bound of LSOM

The role of the eigenvalues *γ*_*i*_ in determining the performance of LSOM can be quantified within a quantum-information framework. The quantum Fisher information—or, equivalently, the reciprocal of the quantum Cramér–Rao bound—sets the maximum achievable accuracy for unbiased estimation of parameters of interest, independent of the specific measurement strategy^7,38,39^.

We derive the quantum Cramér–Rao bound for estimating each Slepian–Pollak coefficient of a confined but otherwise arbitrary optical source profile, under the assumption of optimal measurements of scattered coherent light. From this we obtain a relationship between the resolution, the FOV size *D* and the required total number of scattered photons *P*_tot_ (photon budget) to achieve a desired resolution. The 1D image resolution, Δ, can be approximately expressed as the ratio of the FOV size *D* to the number of Slepian–Pollak coefficients that can be reliably estimated, *N* (refs. ^19,22^) (Supplementary Text 2):

1 $$\Delta \approx \frac{D}{N},$$

where *N* is such that ${P}_{{\rm{tot}}}{|\gamma N|}^{2}\approx 1$.

**Fig. 2:**
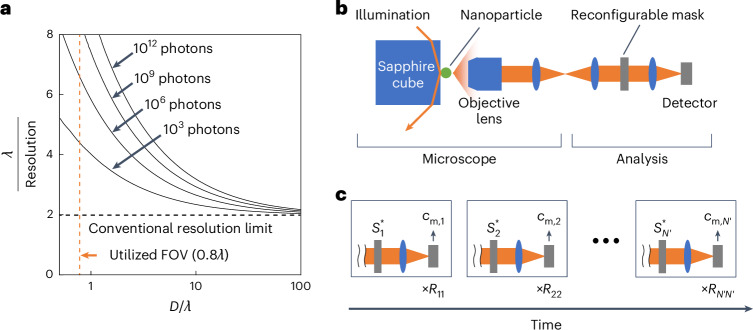
LSOM limits and experimental implementation. **a**, Fundamental bounds on LSOM performance. Optimal resolution as a function of the FOV size *D* and the total number of scattered photons, with representative curves for 10^3^, 10^6^, 10^9^ and 10^12^ photons. The FOV used in the experiments (*D* = 0.8*λ*) is indicated. **b**, Simplified schematic of the experimental LSOM set-up. An object is illuminated using TIR, and the scattered light is collected by a microscope. Slepian–Pollak coefficients at the Fourier plane are measured using a common-path interferometric single-pixel detection scheme with a reconfigurable mask. **c**, Sequential measurement of coefficients. A vector of Slepian–Pollak coefficients is obtained after *N*′ measurements, where the *k*th measurement is repeated *R*_*kk*_ times (*k* = 1, …, *N*′). A mask realizing the complex conjugate (denoted by *) of each Slepian–Pollak function, *S*_*k*_ (*k* = 1, … , *N*′), collimates the corresponding mode.

A graphical representation of equation (1) is shown in Fig. 2a, where the *γ*_*i*_ are linearly interpolated. The extension to 2D imaging is straightforward. For large *D*, achieving resolution far beyond the conventional diffraction limit requires an impractically large photon budget. Conversely, the photon budget needed to reach a target resolution decreases rapidly with *D*. Physically, reducing *D* slows the decay of the effective estimation accuracy for the coefficients *c*_*i*_, enabling high- and low-order coefficients to be estimated with comparable accuracy using a practical number of photons (Supplementary Fig. 1).

### Implementation of the LSOM process

To measure the complex Slepian–Pollak coefficients of the field scattered by a single nano-object (‘Sample preparation’ in the Methods), we built an optical set-up and implemented a method that enabled shot-noise-limited measurements to be achieved (Fig. 2b,c)^40,41^. A simplified schematic of the optical set-up is shown in Fig. 2b: a total internal reflection (TIR) scattering microscope is coupled to a common-path interferometer equipped with a reconfigurable mask (‘Optical set-up’ in the Methods). Active object positioning and drift correction confine the scattering to the FOV and stabilize the measurement (‘Object positioning and drift correction’ in the Methods).

The detected signal arises from interference between the Slepian–Pollak mode of interest (target mode) and a strong Slepian–Pollak mode that, once selected and measured, is used as reference thereafter (reference mode). A reconfigurable digital micromirror device (DMD) placed in the back focal plane of the objective collimates the reference and one target mode at a time along the optical axis while diverting all other modes. A relay lens images these two beams onto the camera sensor. The intensity in the pixel corresponding to the conjugate point of the object centroid encodes the complex coefficient of the target mode; signals from other pixels, which suffer from crosstalk between diverted modes, are discarded. The modulus and phase of each coefficient are obtained from eight submeasurements using distinct DMD masks. The mask set is different for each coefficient. We measure *N*′ coefficients (Fig. 2c) and collect them into an *N*′-by-1 column vector **c**_m_ (‘Measurement of Slepian–Pollak coefficients’ and ‘Selecting an object-centroid pixel and merging LSOM images from multiple illumination directions’ in the Methods).

To reconstruct the optical near-field profile of the object well, it is important to find an accurate estimate, **c**_e_ (*N*-by-1 column vector), of the true Slepian–Pollak coefficients, **c**_o_ (*N*-by-1 column vector) from the measured **c**_m_ (*N*′-by-1 column vector). For a realistic optical system, we have **c**_m_ ≈ *T***c**_o_, where *T* is the *N*′-by-*N* transfer matrix that encodes the optical transfer function of the set-up. Imaging therefore reduces to inverting *T*. We construct a vector linear filter matrix *W* = (*T*^−1^)_mms_—the minimum mean square error solution^42^ of the (pseudo)inverse of *T*—that compensates optical distortions and yields an estimate, **c**_e_ = (*T*^−1^)_mms_**c**_m_ ≈ (*T*^−1^)_mms_*T***c**_o_ ≈ **c**_o_. For the ideal case depicted in Fig. 1b, *T* is diagonal with elements *T*_*ii*_ = *γ*_*i*_. In practice, we experimentally determine (*T*^−1^)_mms_ once, using a single known calibration object—a small dot (for 2D imaging) or a thin line (for 1D imaging) (‘Characterization of the vector linear filter’ in the Methods).

The required photon budget and associated number of repetitions to accurately estimate the *i*th Slepian–Pollak coefficient, **c**_e,*i*_ (*i* = 1, …, *N*), follow from

2 $${P}_{\sin }\frac{{[{\{\mathrm{Re}({T}^{H}T\,)\}}^{-1}]}_{{rr}}}{{[{\{\mathrm{Re}({T}^{H}{RT}\,)\}}^{-1}]}_{{ii}}}\ge 1,$$

where *P*_sin_ is the number of photons measured per submeasurement at the selected pixel, Re denotes the real part of its argument, *H* denotes the Hermitian transpose, *r* is the index of the reference mode and *R* is an *N*′-by-*N*′ diagonal matrix with *R*_*kk*_ equal to the number of repetitions for the *k*th coefficient measurement (*k* = 1, …, *N*′) (Fig. 2c and Supplementary Text 3). *P*_sin_ is chosen to be large enough to satisfy equation (2) without saturating the camera pixels; the condition is met by increasing *R*_*kk*_ for a fixed transfer matrix *T* (‘Required measurement repetitions for accurate filter characterization and coefficient estimation’ in the Methods).

### Experimental demonstration of LSOM

As a proof of principle, we applied LSOM to a range of nanoparticles with a size of <0.8*λ* (*λ* = 638 nm). For each object, we measured and estimated 13 Slepian–Pollak coefficients (*N* = *N*′ = 13) under four different illumination directions, determined by the faces of the sapphire cubes, used as a substrate for the images object. The final LSOM image is obtained through coherent summation of the four reconstructed LSOM images with appropriate phase compensation (‘Selecting an object-centroid pixel and merging LSOM images from multiple illumination directions’ in the Methods). The per-direction LSOM image and raw data are reported in Supplementary Figs. 2 and 3, respectively. The multi-directional illumination not only mitigates artefacts introduced by oblique incidence but also contributes additional resolution enhancement via the in-plane component of the incident wavevector^43,44^.

**Fig. 3:**
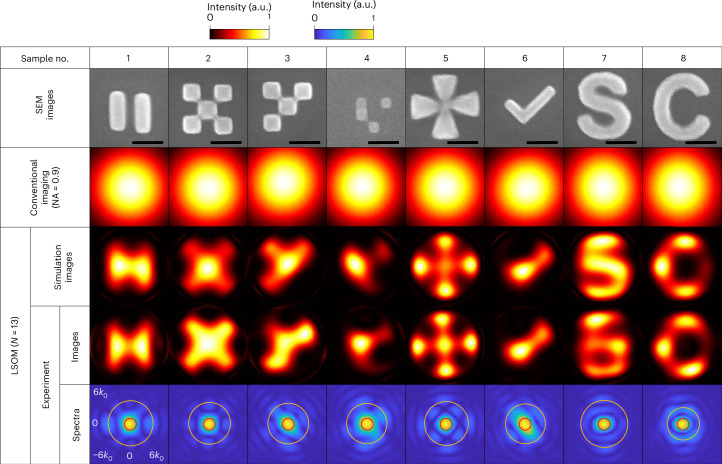
Experimental demonstration of LSOM. Scanning electron microscopy (SEM) images of nanoscale objects with various shapes, sizes and symmetries (top row; scale bars, 200 nm) are compared with ideal conventional coherent Fourier images with NA = 0.9 (second row), simulated LSOM images (third row) and experimental LSOM images (fourth row). LSOM provides a dramatic improvement in resolving power compared with the Fourier imaging. The spatial spectra of the experimental images (fifth row) indicate an effective NA of 3.14, where *k*_0_ = 2*π*/*λ*.

Figure 3 presents the experimental LSOM images of eight platinum (Pt) nanoparticles, fabricated on sapphire cubes using focused electron-beam-induced deposition (FEBID) (‘Sample preparation’ in the Methods). The imaging performance is good for diverse shapes and arrangements, including structures with or without four-fold rotational symmetry (sample numbers 2 and 3), mirror symmetry (compare sample 2 with sample 4), grid-like patterns (compare samples 1–4 with samples 5–8) and curved features (samples 7 and 8). The experimental LSOM images agree well with the numerical simulations (‘Numerical simulations of and calculations of LSOM images’ in the Methods). By contrast, calculated ideal conventional coherent Fourier imaging with a numerical aperture (NA) of 0.9 (the NA of our objective lens) fails to resolve any of these objects (‘Ideal Fourier images and quantification of the LSOM effective numerical aperture’ in the Methods).

**Fig. 4:**
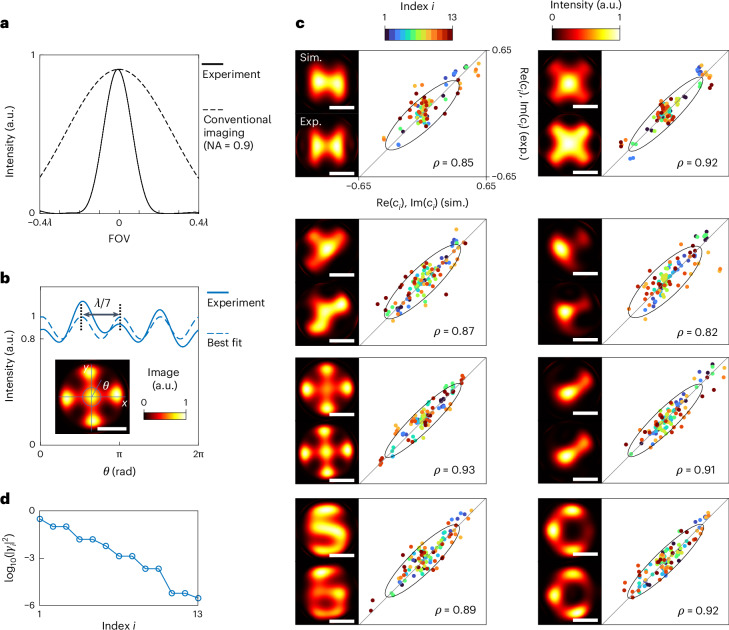
Experimental resolution and Slepian–Pollak coefficient retrieval accuracy. **a**, Representative PSF of LSOM (FWHM ≈ *λ*/6) compared with that of ideal conventional coherent Fourier imaging with NA = 0.9. **b**, Resolution test using a nanoscale Siemens star (sample no. 5 in Fig. 3). The intensity along a circumference with the minimum diameter that satisfies the Rayleigh criterion on average reveals a resolvable centre-to-centre spacing of ~*λ*/7 between neighbouring spokes. *θ* denotes the polar angle of a point on the circumference (inset). Scale bar, 200 nm. **c**, Real (Re) and imaginary (Im) parts of the experimentally (Exp.) measured Slepian–Pollak coefficients for all of the objects (sample numbers 1–8) in Fig. 3 compared with those obtained from simulations (Sim.), showing good estimation accuracy for both strong and weak channels. Each coloured dot corresponds to a different channel; for all channels, the Pearson correlation coefficient, *ρ*, is ≥0.82. The continuous curve in each plot marks the 2*σ* ellipse of the covariance matrix. The labelling in the top left plot also applies to the other seven samples. Scale bars, 200 nm. **d**, Calculated |*γ*_*i*_|^2^ as a function of the mode order *i*, quantifying the decrease in signal strength of Slepian–Pollak modes with increasing order.

We quantify the achieved resolution using several complementary metrics. First, the spatial frequency spectra of the experimental images (Fig. 3, bottom row) show that a substantial portion of the information is well extrapolated outside the limited band (red circle) imposed by the NA of the objective lens. The yellow circles indicate the effective spectral band of the LSOM images, defining an effective NA (‘Ideal Fourier images and quantification of the LSOM effective numerical aperture’ in the Methods). We achieve an effective NA of between 2.45 and 3.57, with an average of 3.14. Supplementary Figure 4 shows 1D LSOM with an effective NA of 3.53. Second, we characterize the overall point spread function (PSF) of the complete imaging chain—from measurement to reconstruction (Supplementary Text 4)^12,23^. Figure 4a compares the PSF of our system with that of ideal Fourier imaging with NA = 0.9. The averaged full-width at half-maximum (FWHM) of the PSFs across positions within the FOV is ~*λ*/6 for 2D imaging and ~*λ*/8 for 1D imaging (Supplementary Fig. 5). Third, we use sample number 5 (shown in Fig. 3) as a nanoscale ‘Siemens Star’. The best-fit sine curve of the intensity profile along a circumference of *λ*/5 diameter fulfils the Rayleigh criterion between two spokes (20% saddle depth or equivalently 10% modulation)^45,46^. This analysis yields a resolvable feature spacing of *λ*/7 (Fig. 4b). Across these three criteria, which comply with ref. ^46^, we consistently obtain a 2D resolution of ~*λ*/7 and a 1D resolution of ~*λ*/8.

Beyond resolution, we directly assess the accuracy of the reconstructed Slepian–Pollak coefficients. Figure 4c compares the simulated and experimentally retrieved 2D coefficients (real and imaginary parts) across all channels and objects, and shows overall excellent agreement (Pearson correlation coefficient *ρ* values of ≥0.82). This holds even for channels in which the normalized energy transfer ratio |*γ*_*i*_/*γ*_1_|^2^ is below 10^−5^ (Fig. 4d) and crosstalk exceeds the true signal (see Supplementary Fig. 6a for an example of the characterized filter). Channel-by-channel comparisons for both 2D and 1D imaging are reported in Supplementary Fig. 7, demonstrating successful information transfer through 1D Slepian–Pollak channels with an energy transfer ratio below 10^−6^.

## Discussion

We have experimentally demonstrated LSOM, a label-free, far-field super-resolution technique that relies solely on prior knowledge of a finite object size, and achieved resolutions of *λ*/7 (2D) and *λ*/8 (1D). In the past, such experimental accomplishment was deemed not feasible because of the extremely high precision required in measuring high-order Slepian–Pollak coefficients of light coherently scattered from nanoscale objects. Our implementation overcomes this barrier, as supported by the reliability of estimations for both low- and high-order complex coefficients (Fig. 4c). The key enabler for this is a shot-noise-limited, sequential, interferometric single-pixel light detection scheme. An important advantage of this single-pixel strategy is in the characterization of the system transfer matrix using a known calibration object: the camera serves as *L* independent single-pixel detectors, effectively providing measurements of the Slepian–Pollak coefficients of the known object at *L* different transverse positions in a single acquisition, which is essential for accurately determining the filter matrix that compensates optical distortions (‘Characterization of the vector linear filter’ in the Methods).

In our experiments we selected 13 Slepian–Pollak modes for 2D imaging and six Slepian–Pollak modes for 1D imaging. Although, in principle, the number of coefficients could be increased by adding further repetitions, maintaining a shot-noise-limited performance becomes progressively more difficult. Supplementary Figure 8 illustrates how the filter performance degrades when attempting 17 coefficients in 2D imaging, even when the number of repetitions is increased as required. A likely limiting factor is residual scattering from surface roughness outside the FOV, a known issue in particle localization^47^ and mass photometry^48^. Such spurious scattering could be mitigated by characterizing the substrate speckle before sample preparation and incorporating this information during coefficient estimation^47,48^.

Although it appears sophisticated, an LSOM set-up can be implemented by simply extending a conventional microscope with a 4*f* relay and a programmable mask. Once assembled, routine operation is straightforward. The usable number of coefficients and the filter matrix can be determined using a standard object by evaluating the residual error in the estimated coefficients. Despite the small FOV, the technique is applicable to many scenarios where nanoscale objects can be isolated or clustered within a confined region, including nanoparticles and nanowires in nanotechnology and particulate pollutants in environmental monitoring. Sample preparation strategies such as solution dilution or deterministic placement^49–51^ via lithographic masking and lift-off^51^ can be used to control the object density and distribution, and minimize background scattering from outside the FOV.

To demonstrate this applicability, we performed the LSOM of commercially available gold (Au) nanoparticles prepared by spin-coating a colloidal solution onto the sapphire cube (‘Sample preparation’ in the Methods), a process that is representative of standard sample preparation for both inorganic and organic nano-objects^51^. By adjusting the solution concentration, we ensured sufficient inter-particle spacing. The resulting LSOM images exhibit no loss of resolution and show resilience to noise from nearby debris (Supplementary Fig. 9). Again, conventional imaging fails to resolve the structures.

In summary, our LSOM technique—underpinned by the analysis of the required measurement repetitions and the construction of an appropriate vector linear filter—demonstrates label-free, far-field super-resolution imaging. The sole prior requirement of a finite object size limit is widely acceptable across many fields and is complementary to other assumptions (such as sparsity) used in earlier super-resolution schemes^8,52^. Beyond imaging, this method is broadly applicable to metrology, spectroscopy and LiDAR (light detection and ranging), wherever accurate recovery of spatial field profiles is required.

## Methods

Full methodological details are provided in Supplementary Methods 1–7.

### Sample preparation

A fine-polished single-crystal sapphire cube is prepared and cleaned using the SC1 (standard clean 1) process with ammonium hydroxide, hydrogen peroxide and deionized water, followed by the SC2 (standard clean 2) process with hydrochloric acid, hydrogen peroxide and deionized water. The Pt nanoparticles (Fig. 3 and Supplementary Fig. 4) were fabricated on the *c* plane of the sapphire cube via FEBID using a C_5_H_4_CH_3_Pt(CH_3_)_3_ precursor. Reference Pt dots and lines used for a filter characterization were patterned in the same step. A 2-nm-thick NbTiN conductive layer was deposited before FEBID to mitigate charging and later oxidized to restore transparency. The nominal Pt thickness was 32 nm.

The Au nanoparticle samples (Supplementary Fig. 9) consisted of two sapphire cubes: one reference cube with electron-beam-lithography-patterned Au dots (80–100 nm diameter, 50 nm thickness) for the filter matrix characterization, and a second cube with spin-coated colloidal Au nanoparticles (Nanopartz), prepared after oxygen plasma treatment for improved wetting. Before preparing the spin-coated colloidal Au nanoparticles, marker arrays were defined using electron-beam lithography (50 × 50 μm pitch, 3 × 3 μm element size), which were used for identifying the nanoparticles. SEM imaging of both Pt and Au samples was performed after optical measurements, following the deposition of a ~3-nm-thick layer of chromium to reduce charging.

### Optical set-up

We utilized a TIR scattering microscope based on a single-crystal sapphire cube (edge length 7.5 mm) that serves both as substrate and TIR prism. Nanoparticles were deposited on the *c* plane of the cube and illuminated with coherent, s-polarized, frequency-stabilized diode lasers (for Pt samples: Cobolt 08-01, HÜBNER Photonics; 80 mW, *λ* = 638 nm, linewidth < 0.1 pm; for Au samples: Lambda Beam Wavelock, RGB Lasersystems; 40 mW, *λ* = 405 nm, linewidth ~0.1 pm). A free-space beam incident at 45° on the cube yields an internal angle of ~66° at the object plane (refractive index ≈ 1.77), ensuring s-polarized TIR illumination. The beam was focused onto a single nanoparticle. Residual aberrations were corrected using a cylindrical lens, resulting in an elliptical spot of ~5 × 12 μm at an incident power of ~10–20 mW.

Scattered light was collected using a high-NA objective lens (MUE13900, Nikon; NA = 0.9) in an inverted microscope (Eclipse Ti, Nikon). The objective’s back focal plane was imaged onto a DMD (DLP500YX, Texas instruments) via a 2*f* relay, with a pupil diameter of ~4.4 mm at the DMD. Complex-amplitude masks were implemented by grouping 4-by-4 micromirrors into super-pixels, operated, for a shifted first diffraction order^53^. The DMD was aligned in Littrow configuration to minimize spatial-mode distortion^54^, and its intrinsic wavefront error was pre-characterized and corrected by applying a phase-conjugated offset to all masks^55^. A scientific CMOS camera (Dhyana 400BSI V2, TUCSEN) was used for light detection. Full details of the set-up are shown in Supplementary Fig. 10.

### Object positioning and drift correction

The sapphire surface (object plane) was first brought into focus using a secondary laser (25-LHP-151, Melles Griot; 5 mW, HeNe) spot reflected from the surface. The centroid of the direct image of the object was aligned to the camera centre pixel using a piezo stage (P-545.3R8S, Physik Instrumente). During acquisition of the coefficients, mechanical drift in the set-up was compensated in real time by alternating between the cycles of drift correction and coefficient measurement every 2–3 s. Full details are provided in Supplementary Method 1 and Supplementary Figs. 11 and 12.

### Measurement of Slepian–Pollak coefficients

To measure the Slepian–Pollak coefficients of the scattered field, the system was operated as a common-path interferometer. A strong Slepian–Pollak mode was chosen as a reference, and each target mode was interfered with this reference in a half-field, multi-step interferometric scheme using the DMD. Eight intensity measurements per mode (with different mask configurations) were used to retrieve the complex coefficient of each target mode. This method can be viewed as a two-arm variant of the process described in ref. ^56^, which requires four submeasurements. The half-field implementation reduces distortions that are present when a single mask is used to superimpose the reference and target modes. Coefficients for *N*′ modes were collected into a column vector **c**_m_. Full details are provided in Supplementary Method 2 and Supplementary Fig. 13.

### Selecting an object-centroid pixel and merging LSOM images from multiple illumination directions

Because oblique illumination shifts the centroid of the direct image relative to the object centroid, a refinement step was implemented to find the object-centroid pixel: several neighbouring pixels were tested, their LSOM images reconstructed and the pixel whose reconstructed image centroid was closest to (*x*, *y*) = (0, 0) in the object coordinate was selected as the object-centroid pixel. The final LSOM images are reconstructed from coherent summation of the LSOM images obtained from the object-centroid pixel for each direction. Full details are provided in Supplementary Method 3 and Supplementary Fig. 12.

### Characterization of the vector linear filter

The filter matrix *W* = (*T*^−1^)_mms_ was experimentally determined using a single known calibration object (an 80 nm dot for 2D imaging or an 80 nm line for 1D imaging) and exploiting the conjugate relation between object and camera planes: each camera pixel corresponds to a distinct transverse shift of the calibration object. Thus, *L* ≈ 100 independent realizations of **c**_m_ were obtained in a single acquisition by reading neighbouring pixels, whereas the corresponding **c**_o_ were computed by modelling the calibration object as ideal. Full details are provided in Supplementary Method 4.

### Required measurement repetitions for accurate filter characterization and coefficient estimation

The required number of measurement repetitions for each coefficient, encoded in a diagonal repetition matrix *R*, was obtained from an analytic expression for *W* under shot-noise-limited conditions and refined iteratively until obtaining convergence of *W* and its residual error. This ensured a sufficient signal-to-noise ratio across all measured channels. Full details are provided in Supplementary Method 5.

### Numerical simulations of and calculations of LSOM images

LSOM images were computed from finite-difference time-domain simulations (Ansys Lumerical) followed by Fourier optics propagation^57^. A 6 × 6 × 2 μm domain with total-field scattered-field plane-wave illumination at ~66° was used. Scattered fields were transformed to the far field and then mapped onto the back focal plane of an NA-limited objective lens. After applying the same Slepian–Pollak masks and reconstruction procedure as in the experiment, simulated coefficient vectors **c**_m_ were obtained and processed through the vector linear filter matrix to yield **c**_e_. The vector linear filter matrix was also constructed by mimicking the experimental process described in ‘Characterization of the vector linear filter’ in the Methods. Full details are provided in Supplementary Method 6.

### Ideal Fourier images and quantification of the LSOM effective numerical aperture

Ideal coherent Fourier images were generated from binarized SEM images (assumed to scalar near-field amplitude profiles with uniform phase) by sequentially performing Fourier transformation, low-pass filtering (corresponding to applying a lens NA) and inverse Fourier transformation.

Effective NAs were quantified by comparing both the reconstructed LSOM images and the ideal coherent Fourier images (with varying NA) against the binarized SEM images. After aligning and interpolating both datasets, the normalized fidelity metric was computed. The effective NA was defined as that of the Fourier image whose fidelity best matched the fidelity of the LSOM reconstruction. Full details are provided in Supplementary Method 7.

## Online content

Any methods, additional references, Nature Portfolio reporting summaries, source data, extended data, supplementary information, acknowledgements, peer review information; details of author contributions and competing interests; and statements of data and code availability are available at 10.1038/s41566-025-01839-2.

## Supplementary information

Supplementary Information Supplementary Methods 1–7, Texts 1–4, Figs. 1–14 and refs. 1–9.

## Data Availability

The data that support the findings of this study are openly available from the NTU research data repository DR-NTU (Data) at 10.21979/N9/RERFQJ.
