# Supplementary material for: Super-resolution imaging of limited-size objects
Source: Nat Photonics. 2026 Feb 16;20(4):421–7. doi: 10.1038/s41566-025-01839-2 (PMC13061642; doi:10.1038/s41566-025-01839-2)
Supplement: Supplementary file 1 — Supplementary Methods 1–7, Texts 1–4, Figs. 1–14 and refs. 1–9. [file 41566_2025_1839_MOESM1_ESM.pdf]

nature photonics

Supplementary information https://doi.org/10.1038/s41566-025-01839-2

Super-resolution imaging of limited-size objects

In the format provided by the authors and unedited

Contents

Supplementary Methods 2

1. Object positioning and drift correction. 2
2. Measurement of Slepian-Pollak coefficients. 3
3. Selecting the object-centroid pixel and merging LSOM images from multiple illumination directions. 4
4. Characterization of the vector linear filter. 5
5. Required measurement repetitions for accurate filter characterization and coefficient estimation. 6
6. Simulations and calculations of LSOM images. 8
7. Ideal Fourier images and quantification of LSOM effective numerical aperture. 9

Supplementary Text 11

1. Reciprocal relationship between superoscillatory hotspot generation and LSOM. 11
2. Fundamental resolution limit of LSOM from the quantum Cramér–Rao bound. 13
3. Classical Cramér–Rao bound and signal-to-noise ratio for our measurement scheme. 16
4. Overall point spread function. 20

Supplementary Figures (S1–14) 23

Supplementary References 37

Supplementary Methods

1. Object positioning and drift correction.

Before the coefficient measurements, the sapphire surface (object plane) is brought into focus. The z coordinate of the surface is determined via maximum likelihood estimation (MLE) using the modified direct image of a secondary laser beam, reflected from the cube surface (Supplementary Fig. S10c). To achieve accurate z-localization, we modify the direct image of the secondary laser beam spot using a dedicated DMD mask, because the unmodified image of a point-like object is unsuitable for z-localization[1]. The mask (Supplementary Fig. S11a) spatially multiplexes two patterns: the outer region changes the orbital angular momentum by +2 order, while the inner region does not. Calculations show that the modified image of a point-like object (Supplementary Fig. S11b) yields per-photon Cramér–Rao bounds of 0.1λ, 0.14λ, and 0.32λ (in terms of standard deviation) for x-, y-, and z-localization, respectively, which is adequate for alignment. Probability distributions (modified images) as a function of z are pre-recorded for MLE. Then, any nonzero z offset is corrected with the piezo stage.

Next, the centroid of the intensity-truncated direct image is computed by evaluating $\min[I(x_{\mathrm{cam}}, y_{\mathrm{cam}}), 0.1\cdot\max[I(x_{\mathrm{cam}}, y_{\mathrm{cam}})]]$, where $I(x_{\mathrm{cam}}, y_{\mathrm{cam}})$ is the intensity profile of the direct image. The stage is then adjusted so that this centroid coincides with the *camera center pixel*, the 1025th pixel in both horizontal and vertical indices of the 2048-by-2048 camera sensor array (Supplementary Fig. S12a). It shall be noted that the centroid of the intensity-truncated direct image may not coincide with the conjugate point of the object centroid along to the oblique incidence illumination. This requires additional compensation by the procedure outlined in Supplementary Methods 3.

During coefficient measurements, mechanical drifts of the optical setup are actively compensated, so that measurements remain limited by shot noise, rather than drift. Drift-compensation and data-acquisition cycles alternate every 2–3 seconds. Although different components induce different distortions in the scattering pattern, the net effect is well approximated as an effective object displacement. All x-, y-, and z-displacements are determined via MLE from modified direct images of the object. Probability distributions for z-displacements are pre-recorded, and those for x- and y-displacements (for each z-displacement) are computed immediately after object positioning, before the drift compensation.

Supplementary Fig. S11c shows that the object position is well maintained throughout the measurements; Supplementary Fig. S11d shows that the total drift compensated by the stage is a few hundred nanometers (inferred from applied voltages). The stage is operated in open-loop mode to avoid vibrations associated with closed-loop control.

2. Measurement of Slepian-Pollak coefficients.

Here we provide further details on the measurement of $\mathbf{c}_\mathrm{m}$, as introduced in the main text. We first select a sufficiently strong Slepian–Pollak mode—the strongest among the three modes with largest $|\gamma_i|$—and use it as a reference for all subsequent measurements. We then measure the interference signal between this reference mode and each target mode $k = 1, \ldots, N'$.

Rather than using a single mask that superposes the reference and target mode profiles (which would introduce additional distortions), we employ a multi-step scheme: (1) Measure light intensity at the camera, I_a, with a DMD mask implementing the reference mode profile on one half of the DMD (denoted here as left half) and zero-amplitude on the other half (denoted here as right half). (2) Measure light intensity at the camera, I_b, with a DMD mask implementing the reference mode profile on the right half and zero-amplitude on the left half. (3) Retrieve the phase retardance ϕ_ba between these two cases by applying four masks that generate different relative phase shifts between the left and right halves of the reference mode profile. (4) Apply the reference mode on the left half and the kth target mode on the right half with four relative phase shifts (0, 0.5π, π, and 1.5π), and measure the corresponding intensities $I_{\mathrm{a},v}^{(k)}$ ($v = 1$–4). (5) Swap the halves and repeat step 4, obtaining $I_{\mathrm{b},v}^{(k)}$ ($v = 1$–4). Steps (1)–(3) are performed once at the beginning. The complex amplitude of the kth target coefficient is then retrieved from these eight intensities using

$$c_{\mathrm{m},k} = \sum_{v=1}^{4} \frac{1}{4} \left[\frac{(-i)^v I_{\mathrm{a},v}^{(k)}}{\sqrt{I_\mathrm{a}}} + e^{i\phi_\mathrm{ba}} \frac{(-i)^v I_{\mathrm{b},v}^{(k)}}{\sqrt{I_\mathrm{b}}} \right]. \tag{S1}$$

The set of measured coefficients $c_{m,k}$ (k = 1, …, N') forms the column vector $\mathbf{c}_m$. The corresponding DMD masks are shown in Supplementary Fig. S13.

Because the interference pattern is recorded as an image, each camera pixel provides its own coefficient vector $\mathbf{c}_m$. Applying the filter matrix $(T^{-1})_{mms}$ pixel-by-pixel yields a spatial map of estimated coefficients $\mathbf{c}_e = (T^{-1})_{mms} \cdot \mathbf{c}_m$ (Supplementary Fig. S12b). The characterization of $(T^{-1})_{mms}$ is described in Supplementary Methods 4.

3. Selecting the object-centroid pixel and merging LSOM images from multiple illumination directions.

Accurate merging of LSOM images obtained under different illumination directions requires identifying the exact object position (represented by its centroid). To achieve this, we identify the *object-centroid pixel*: the camera pixel corresponding to the conjugate point of the object centroid (Supplementary Fig. S12c). The positioning procedure in Supplementary Methods 1 ensures that this point lies near the camera center, but does not identify the exact pixel because, under oblique illumination, the centroid of an image can be shifted relative to the true object centroid along the illumination direction (but not along the orthogonal direction) (main text Ref. 43–44).

To refine the centroid position, we exploit the higher resolution of LSOM images. For each illumination direction (aligned with either the x or y axis), we select five pixels near the *camera center pixel*, generate five vectors $\mathbf{c}_e$ from the measured $\mathbf{c}_m$, reconstruct five LSOM images, and compute the centroid of each image. The pixel whose reconstructed image centroid is closest to (x, y) = (0, 0) is designated as the *object-centroid pixel*. To reduce the influence of distortions from oblique illumination and noise, we threshold the reconstructed LSOM images (i.e. $\left|\sum_{j=1}^{N} c_{e,j} S_j(\mathbf{r})\right|^2$) and compute centroids from the truncated intensity distribution (i.e. $\min\left[\left|\sum_{j=1}^{N} c_{e,j} S_j(\mathbf{r})\right|^2, f_{\max}\right]$, where $f_{\max} = 0.1 \cdot \max_{\forall \mathbf{r}}\left[\left|\sum_{j=1}^{N} c_{e,j} S_j(\mathbf{r})\right|^2\right]$). The five candidate pixels are chosen along the axis aligned with the illumination direction; no refinement is needed along the orthogonal axis.

To form the final LSOM image, we coherently combine reconstructions obtained from different illumination directions (four for 2D imaging, two for 1D imaging) using different facets of the sapphire cube. The final image is the squared magnitude of the coherent sum of the sets of Slepian-Pollak coefficients, with total internal reflection (TIR) illumination phases compensated, as expressed in Eq. (S2)

$$\text{Image} = \left| \sum_{l=1}^{4\ (\text{or}\ 2)} \left[e^{-i(\mathbf{k}_{\text{TIR},l}\cdot\mathbf{r}+\phi_l)} \sum_{j=1}^{N} c_{\text{e},j} S_j(\mathbf{r}) \right] \right|^2 \tag{S2}$$

where $\mathbf{k}_{\text{TIR},l}$ is the wavevector of lth illumination and $\phi_l = \angle\left[\int_{|\mathbf{r}|\le R_{\text{O}}} \left(\sum_{k=1}^{N} \mathbf{c}_{\text{e},l}(k) S_k(\mathbf{r}) \right) d\mathbf{r} \right]$ is a lth global phase correction factor ($l = 4$ for 2D imaging, $l = 2$ for 1D imaging).

4. Characterization of the vector linear filter.

In general, a vector linear filter is an N-by-N' matrix. Here, as in our experiments, we consider $N'=N$. For a real optical system, a measured coefficient vector satisfies $\mathbf{c}_{\text{m}} = T\mathbf{c}_{\text{o}}$ where T is the N-by-N transfer matrix and $\mathbf{c}_{\text{o}}$ is a N-by-1 vector of true Slepian–Pollak coefficients of the effective source (optical near-field under a given illumination). An estimated N-by-1 vector, $\mathbf{c}_{\text{e}} = W\mathbf{c}_{\text{m}} = (T^{-1})_{\text{mms}}\cdot\mathbf{c}_{\text{m}}$ is then a solution to the LSOM problem. Here $\mathbf{c}_{\text{e}}$ is a vector linear minimum mean square error (VLMMSE) estimator—the vector linear estimator minimizing the mean square error between estimated and true vectors—and $W=(T^{-1})_{\text{mms}}$ is the N-by-N VLMMSE filter. W is characterized via Eq. (S3),

$$W = \Omega_{\text{om}} \Omega_{\text{mm}}^{-1} \tag{S3}$$

where $\Omega_{\text{om}} = C_{\text{o}} C_{\text{m}}^{H}$, $\Omega_{\text{mm}} = C_{\text{m}} C_{\text{m}}^{H}$, $C_{\text{m}} = [\mathbf{c}_{\text{m}}^{(1)}, \mathbf{c}_{\text{m}}^{(2)}, \ldots, \mathbf{c}_{\text{m}}^{(L)}]$, $C_{\text{o}} = [\mathbf{c}_{\text{o}}^{(1)}, \mathbf{c}_{\text{o}}^{(2)}, \ldots, \mathbf{c}_{\text{o}}^{(L)}]$, $\mathbf{c}_{\text{m}}^{(i)}$ is a N-by-1 column vector of the measured coefficients for ith illuminated object ($i = 1, \ldots, L$),

and $\mathbf{c}_\mathrm{o}^{(i)}$ is a N-by-1 column vector of the true coefficients for ith illuminated object ($i = 1, \ldots, L$), and H denotes the Hermitian transpose, respectively (main text Ref. 42).

A single object at a fixed position is insufficient to fully characterize W. At least $L \geq N$ distinct known objects are required, yielding L unique pairs of $\mathbf{c}_\mathrm{o}$ and $\mathbf{c}_\mathrm{m}$. Substituting the matrix identities $\Omega_\mathrm{om} = C_\mathrm{o} C_\mathrm{m}^H$ and $\Omega_\mathrm{mm} = C_\mathrm{m} C_\mathrm{m}^H$ into Eq. (S3) yields $W = C_\mathrm{o} C_\mathrm{m}^H (C_\mathrm{m}^H)^{+} (C_\mathrm{m})^{+}$. This shows that if $L < N$, C_o and C_m become tall matrices, and W becomes rank-deficient, so matrix inversion, even in absence of noise, cannot fully compensate system distortions.

Two alternative strategies can be pursued to avoid fabricating L physical distinct objects: use a single known object and (1) shift it physically in the object plane, or (2) exploit virtual shifts in the camera sensor plane. Because the camera sensor plane and the object plane are conjugate, each camera pixel maps to a specific transverse shift of the object, and vice versa. We use standard objects, consisting of an 80 nm dot and an 80 nm line as 2D and 1D calibration samples, respectively. Small dots or lines at different transverse positions span a suitable set of objects, as the singular values of the N-by-N autocorrelation matrix of all $\mathbf{c}_\mathrm{o}$, Ω_oo, are sufficiently large. The matrix C_o collects L unique true coefficient vectors $\mathbf{c}_\mathrm{o}$ corresponding to dots (or lines) at L positions. These coefficients, and $\Omega_\mathrm{oo} = C_\mathrm{o} C_\mathrm{o}^H$, are computed by modelling the calibration object as an ideal dot (or line). We adopt strategy (2) to measure L unique $\mathbf{c}_\mathrm{m}$ experimentally: we position the calibration object at a fixed position in the object plane and collect coefficient vectors from L neighbouring camera pixels. Because each N-by-1 vector $\mathbf{c}_\mathrm{m}$ can be retrieved from a single pixel, C_m can be constructed from ~100 pixels near the *camera center pixel* in a single acquisition.

5. Required measurement repetitions for accurate filter characterization and coefficient estimation.

The filter accuracy is limited by shot noise in the calibration measurements, which can be controlled by number of measurements repetitions. In the following, we find the required number of repetitions. In the presence of measurement noise, the measured coefficient vector can be written as $\mathbf{c}_\mathrm{m} = T\mathbf{c}_\mathrm{o} + \mathbf{c}_\mathrm{n}$, where $\mathbf{c}_\mathrm{n}$ is an N-by-1 noise vector. Because the expectation value of each element of $\mathbf{c}_\mathrm{n}$ is zero, $\Omega_\mathrm{om} = \Omega_\mathrm{oo} T^H$ and $\Omega_\mathrm{mm} = T \Omega_\mathrm{oo} T^H + \Omega_\mathrm{nn}$ for sufficiently

large L, where Ω_{nn} is the N-by-N autocorrelation matrix of $\mathbf{c}_{\text{n}}$. Assuming $\left\|T\Omega_{\text{oo}}T^H\right\|_{\text{F}} \gg \left\|\Omega_{\text{nn}}\right\|_{\text{F}}$ where $\|A\|_{\text{F}}$ is the Frobenius norm of A, we can approximate $\Omega_{\text{mm}}^{-1} \approx (T^{-1})^H \Omega_{\text{oo}}^{-1} T^{-1} + (T^{-1})^H \Omega_{\text{oo}}^{-1} T^{-1} \Omega_{\text{nn}} (T^{-1})^H \Omega_{\text{oo}}^{-1} T^{-1}$. Substituting into Eq. (S3) yields the approximation

$$W \approx W_0 \left(I + \Omega_{\text{nn}} W_0^H \Omega_{\text{oo}}^{-1} W_0 \right) \tag{S4}$$

where $W_0 = T^{-1}$ is the ideal filter matrix and I is an N-by-N identity matrix.

The autocorrelation matrix of $\mathbf{c}_{\text{n}}$ can be written as $\Omega_{\text{nn}} = \sigma_{\text{n}}^2 R^{-1} = 2R^{-1}$, where σ_{n}^2 is the single-measurement noise variance ($\sigma_{\text{n}}^2 = 2$ for a measured random vector X_k, see Supplementary Text 3), and R is the diagonal repetition matrix whose kth diagonal component, R_{kk}, is the number of repetitions for kth coefficient measurement . Therefore, we require R to satisfy these conditions

$$\begin{cases} \partial \left\| W_0 \left(I + 2R^{-1} W_0^H \Omega_{\text{oo}}^{-1} W_0 \right) \right\|_{\text{F}} \Big/ \partial R_{kk} = \text{constant for all } k \\ \text{and} \\ \left\| 2R^{-1} W_0^H \Omega_{\text{oo}}^{-1} W_0 \right\|_{\text{F}} = \varepsilon \left\| I \right\|_{\text{F}} \end{cases} \tag{S5}$$

where ε is an error tolerance that should be kept small (e.g. in our case, $\varepsilon = 0.1$). We record ~1500 photons per frame per pixel, most of which are generated by the reference mode (the first Slepian–Pollak mode for the calibration object). Therefore, $W_0^H \Omega_{\text{oo}}^{-1} W_0$ can be normalized so that its (1,1) component becomes 1/1500 by assuming $|T_{11}| \approx 1 \gg |T_{1p}|$ ($p = 2, \ldots N$) and using $\Omega_{\text{mm},11} \approx \Omega_{\text{oo},11} \approx 1500$.

Because Ω_{oo} is known, Eq. (S5) provides an analytical solution for R, once W_0 is determined. In practice, we use an iterative procedure: starting from an initial guess for R, we measure W, and insert it into Eq. (S5) as a proxy for W_0, compute an updated R, remeasure W and repeat until convergence. Supplementary Fig. S6a shows the experimentally characterized W; Supplementary Fig. S6b compares the characterized R_{kk} values to those used in 2D imaging. The small residual error in the estimated coefficients, averaged over L pixels (i.e.

$\left[(C_\text{o}-WC_\text{m})(C_\text{o}-WC_\text{m})^H\right]_{ii}/\left[C_\text{o}C_\text{o}^H\right]_{ii}$), shown in Supplementary Fig. S6c confirms good filter performance.

The expected signal-to-noise ratio for the ith coefficients for a given R, $\text{SNR}_{R,i}$, is computed using Eq. (S34) in Supplementary Text 3 under the assumption $T=W^{-1}$, and is sufficiently high for all i (Supplementary Fig. S6d). Therefore, it can be confirmed that R is not only sufficient for the accurate characterization of the filter but also for the estimation of the Slepian–Pollak coefficient of unknown objects.

6. Simulations and calculations of LSOM images.

Simulated LSOM images (Fig. 3, Supplementary Fig. S4, and Supplementary Fig. S9) are generated using finite-difference time-domain (FDTD) simulations followed by Fourier-optics calculations. The simulation geometry is shown in Supplementary Figs. S14a–c. The FDTD computational domain (excluding perfectly matched layers) is 6 μm × 6 μm × 2 μm. The mesh size is 10 nm near the objects and 20–30 nm elsewhere. TIR illumination at ~66° is implemented using a total-field scattered-field (TFSF) source. The TFSF source is a plane wave that allows monitoring of scattered fields only, by subtracting the incident wave outside the source region (in 3D simulations this is implemented by a box). A 15 fs pulse is used; in linear FDTD simulation, the pulse duration does not affect the frequency-domain response.

The LSOM images are obtained as follows. (1) Electric (**E**) and magnetic (**H**) field vectors of the scattered light are extracted on a transverse plane (6 μm × 6 μm) located 10 nm above the TFSF source region. (2) Far-field **E** and **H** on a hemisphere surface are computed with the built-in near-to-far-field transformation (Supplementary Fig. S14d). In the far field, $\mathbf{E}\cdot\mathbf{n}$ and $\mathbf{H}\cdot\mathbf{n}$ are negligible and **H** is proportional to $\mathbf{n}\times\mathbf{E}$, so only E_θ (= $\mathbf{E}\cdot\boldsymbol{\theta}$) and E_ϕ (= $\mathbf{E}\cdot\boldsymbol{\phi}$) are needed. (3) The x-component of the electric field at the back focal plane (x'-y' plane) is computed as $E_x(x', y') = \cos(\phi)\cdot E_\theta - \sin(\phi)\cdot E_\phi$. The spatial coordinates (x', y') are proportional to spatial frequencies (k_x, k_y), with $x' = \text{NA}\cdot k_0\cdot\sin(\theta)\cdot\cos(\phi)$ and $y' = \text{NA}\cdot k_0\cdot\sin(\theta)\cdot\sin(\phi)$, where NA is the objective lens numerical aperture and k_0 is the free-space wavevector (Supplementary Fig. S14e). We use only E_x in both simulations and experiments. (4) The reconstruction problem (for each illumination direction) reduces to an inverse problem for a scalar field measured at the camera. The field at the camera sensor plane is computed using Fourier-optic calculations

following the formalism in main text Ref. 57 (chapter 4.1), by applying the transfer functions of free space, the lens, and the ideal masks for each Slepian–Pollak mode. (5) The complex field at the center of the camera sensor plane for the ith Slepian–Pollak mask is taken as the ith coefficient c_i. The N-by-1 column vector $\mathbf{c}_m$ is constructed by collecting c_i (i = 1, …, N). (6) The transfer matrix T is obtained using the same procedure as in Supplementary Methods 4, simulating a small dot. Although the simulated T is closer to diagonal than the experimental matrix, it still has non-zero off-diagonal elements. (7) For all the other objects, we obtain the estimated coefficients from $\mathbf{c}_e = (T^{-1})_{mms} \cdot \mathbf{c}_m$.

For each object, four simulations corresponding to four illumination directions are performed and combined according to Eq. (2) in Supplementary Methods 3, as in the experiment.

The effective electric permittivities of Pt and Au are modelled as follows. Pt nanoparticles fabricated by focused electron beam induced deposition (Methods 1 in the main text) have ~50% metal content (by mass) and optical properties different from bulk Pt. We use a Maxwell–Garnett effective medium model for the permittivity, ε_{MG}: $\varepsilon_{MG} = \varepsilon_d + 3\varepsilon_d \cdot c_m \cdot (\varepsilon_m - \varepsilon_d) \cdot [\varepsilon_m + 2\varepsilon_d - c_m \cdot (\varepsilon_m - \varepsilon_d)]^{-1}$ where $\varepsilon_d \approx 2+3i$ (assuming graphite), $\varepsilon_m \approx -21+22i$ (assuming pure platinum), and a volume filling fraction c_m = 0.1 (considering the density of graphite and platinum), yielding $\varepsilon_{MG} \approx 2.2 + 4.1i$ (at λ = 638 nm). The electric permittivity of gold is taken from Johnson and Christy; Ansys Lumerical's proprietary multi-coefficient material model is used to fit the tabulated data.

7. Ideal Fourier images and quantification of LSOM effective numerical aperture.

To calculate an ideal coherent Fourier image, we treat the binarized SEM image as the amplitude of an effective scalar near-field with uniform phase. A Fourier transform of the binarized SEM image is computed (MATLAB), a low-pass filter (corresponding to a lens NA) is applied, and an inverse Fourier transform is performed. The intensity image is then obtained by taking the squared magnitude of the resulting field.

The effective numerical aperture NA_{eff} for each LSOM image is obtained as follows. (1) A 510 nm-by-510 nm region (for Fig. 3 and Supplementary Fig. S4) or a 506 nm-by-506 nm region (for Supplementary Fig. S9) is cropped from the as-acquired SEM image. (2) The cropped image is binarized using MATLAB's imbinarize function. (3) A corresponding 500 nm-by-500

nm region is cropped from the reconstructed LSOM image, with center (x_0, y_0) chosen to visually match the binarized SEM image. (4) The LSOM image is interpolated to the same pixel count as the binarized SEM image. (5) The fidelity, $\mathrm{Fid}(x_0, y_0)$ between the binarized SEM and the reconstructed LSOM images is computed as

$$\mathrm{Fid}(x_0, y_0) = \sum_{\text{All pixels}} \left(\frac{I_{\text{bSEM}}}{\sqrt{\sum_{\text{All pixels}} I_{\text{bSEM}}^2}} \right) \left(\frac{I_{\text{Re}}(x_0, y_0)}{\sqrt{\sum_{\text{All pixels}} I_{\text{Re}}^2(x_0, y_0)}} \right) \tag{S6}$$

where I_{bSEM} and I_{Re} are pixel values of the binarized SEM and reconstructed LSOM images, respectively. The normalization ensures Fid $\leq$ 1. (6) The fidelity is maximized over small spatial shifts of the LSOM image: $\mathrm{Fid}_{\max} = \max_{i,j} \left[\mathrm{Fid}\left(x_0 \pm i, y_0 \pm j \right) \right]$ where i and j are the shifts, in number of pixels. (7) Fidelities are also computed for ideal Fourier images with varying NA over unity. (8) The NA_{eff} of a LSOM image is defined as the NA for which the fidelity of the ideal Fourier image most closely matches that of the LSOM image.

Supplementary Text

1. Reciprocal relationship between superoscillatory hotspot generation and LSOM.

In Fig. 1 of the main text, we consider wave propagation between two confined regions. Equivalently, this can be viewed as an optical system with confined sets of multimode input and output ports; any radiation outside these regions is treated as loss. In Fig. 1b in the main text (forward propagation), the input source is localized at the object plane and the output wave is observed in the Fourier plane. In this configuration, Slepian–Pollak functions form the normal modes propagation because they are eigenfunctions of the finite Fourier transform (main text Ref. 29–30):

$$\int_{|x|\le D/2} G(x',x)S_i(x)dx = \gamma_i S_i(x') \quad (\text{for } |x'| \le K/2) \tag{S7}$$

where G, S_i, and γ_i are the Green's function, the ith Slepian–Pollak function (i = 1, 2, …), and the complex amplitude transfer ratio for that mode, respectively. The Slepian–Pollak functions satisfy the orthonormality condition

$$\int_{|x|\le D/2} S_i(x)S_j(x)dx = \delta_{ij} \quad \text{and} \quad \int_{|x'|\le K/2} S_i(x')S_j(x')dx' = \delta_{ij} \,. \tag{S8}$$

Multiplying Eq. (S7) by $S_j(x')$ and integrating over x', then applying Eq. (S8), yields

$$\int_{|x'|\le K/2}\int_{|x|\le D/2} G(x',x)S_i(x)dxS_j(x')dx' = \gamma_i \delta_{ij} \,. \tag{S9}$$

Using reciprocity of the Green's function[2], $G(x',x) = G(x,x')$, and the orthonormality of the Slepian–Pollak functions, we obtain

$$\int_{|x|\le D/2}\left[\int_{|x'|\le K/2} G(x,x')S_j(x')dx'\right]S_i(x)dx = \gamma_i\delta_{ij} = \gamma_i\int_{|x|\le D/2} S_j(x)S_i(x)dx\,. \qquad \text{(S10)}$$

Therefore,

$$\int_{|x'|\le K/2} G(x,x')S_j(x')dx' = \gamma_j S_j(x) \quad (\text{for } |x|\le D/2)\,. \qquad \text{(S11)}$$

Equation (S11) shows that Slepian–Pollak functions are also the normal modes for the backward propagation configuration (Fig. 1a in the main text). In modal form, the scattering amplitude $f(S_i, S_j)$, satisfies the Helmholtz reciprocity relation[3], $f(S_i, S_j) = f(-S_j, -S_i) = \gamma_i\delta_{ij}$, which follows directly from Eqs. (S7) and (S11) and the reciprocity of G.

Following the definition in Supplementary Ref. 4, two optical configurations are reciprocal configurations as reciprocal configurations if the input profile in one setup coincides with the output profile in the other. In the superoscillatory hotspot generation (Fig. 1a in the main text), an input field $\sum_i \gamma^{-1}c_i S_i(x')$ at the Fourier plane produces a superoscillatory field $\sum_i c_i S_i(x)$ in the object plane following Eq. (S11). In LSOM (Fig. 1b in the main text), the latter field acts as the the source, and its radiation in the Fourier plane is described by $\sum_i \gamma_i c_i S_i(x')$ following Eq. (S7). Thus, the two configurations form reciprocal configurations.

For an image reconstruction, suppose a field $\sum_i c_{\mathrm{m},i} S_i(x')$ is measured in the Fourier plane while the object-plane source $\sum_i c_{\mathrm{o},i} S_i(x)$ is unknown. The imaging task is to determine $c_{\mathrm{o},i}$ from the Fredholm integral equation

$$\int_{|x|\le D/2} G(x',x)\left[\sum_i c_{\mathrm{o},i} S_i(x)\right]dx = \sum_i c_{\mathrm{m},i} S_i(x') \quad (\text{for } |x'|\le K/2)\,, \qquad \text{(S12)}$$

with given G by Eq. (S7). By substituting Eq. (S7) into Eq. (S12), we obtain $\sum_i \gamma_i c_{\mathrm{o},i} S_i(x') = \sum_i c_{\mathrm{m},i} S_i(x')$. By applying the orthonormality, it can be concluded that $c_{\mathrm{o},i}$ =

$c_{m,i}/\gamma_i$ for all i. Thus, in the ideal case, recovering the Slepian–Pollak coefficients is straightforward once γ_i is known.

2. Fundamental resolution limit of LSOM from the quantum Cramér–Rao bound.

We first derive the N-by-N quantum Fisher information matrix, $\mathcal{I}(c_1,...,c_N)$, and the corresponding quantum Cramér–Rao bounds for simultaneous estimation of N Slepian–Pollak coefficients of the source profile, using measurements of the coefficients of the scattered field following Supplementary Ref. 5–6 and main text Ref. 38.

For multiparameter estimation, the (p, q) element of the quantum Fisher information matrix is defined as[5]

$$\mathcal{I}_{pq} = \frac{1}{2}\mathrm{Tr}\left[\rho\left(L_p L_q + L_q L_p\right)\right] \tag{S13}$$

where ρ is the density operator and L_r ($r = p$, q) is the symmetric logarithmic derivative with respect to the parameter θ_r. L_r is implicitly given by

$$\rho L_r + L_r \rho = 2\partial_r \rho \tag{S14}$$

where $\partial_r[A] = \partial[A]/\partial\theta_r$. We model the scattered field as a separable pure N-mode coherent state (main text Ref. 38), $\rho = |\psi\rangle\langle\psi|$ where $|\psi\rangle = |\alpha_1\rangle\otimes\cdots\otimes|\alpha_N\rangle$ and $|\alpha_k\rangle$ is the coherent state of the kth single mode with amplitude α_k equal to the complex field coefficient. Using the condition for the pure state, the condition for unitary transformation (i.e. $\partial_r\left(\langle\psi|\psi\rangle\right) = 0$), and its consequences (i.e. $\mathrm{Re}\left(\langle\psi|\partial_r\psi\rangle\right) = 0$ and $\langle\psi|L_r|\psi\rangle = 0$)[6], the equation $\left(\rho L_p + L_p\rho\right)\left(\rho L_q + L_q\rho\right) + \left(\rho L_q + L_q\rho\right)\left(\rho L_p + L_p\rho\right) = 4\partial_p\rho\cdot\partial_q\rho + 4\partial_q\rho\cdot\partial_p\rho$ yields

$$\mathcal{I}_{pq} = \frac{1}{2}\langle\psi|L_pL_q + L_qL_p|\psi\rangle = 4\,\mathrm{Re}\left[\langle\partial_p\psi|\partial_q\psi\rangle\right] - 4\,\mathrm{Re}\left[\langle\psi|\partial_p\psi\rangle\langle\partial_q\psi|\psi\rangle\right]. \quad \text{(S15)}$$

Using the condition for the separable state, Eq. (S15) becomes

$$\begin{aligned}\mathcal{I}_{pq} = 4\,\mathrm{Re}&\left(\sum_{k=1}^{N}\langle\partial_p\alpha_k|\partial_q\alpha_k\rangle + \sum_{k,l=1(k\neq l)}^{N}\langle\partial_p\alpha_k|\alpha_k\rangle\langle\alpha_l|\partial_q\alpha_l\rangle\right) \\ &-4\,\mathrm{Re}\left(\sum_{k,l=1}^{N}\langle\alpha_k|\partial_p\alpha_k\rangle\langle\partial_q\alpha_l|\alpha_l\rangle\right)\end{aligned}, \quad \text{(S16)}$$

which can be further reduced to

$$\mathcal{I}_{pq} = 4\sum_{k=1}^{N}\left[\mathrm{Re}\left(\langle\partial_p\alpha_k|\partial_q\alpha_k\rangle\right) - \mathrm{Re}\left(\langle\alpha_k|\partial_p\alpha_k\rangle\langle\partial_q\alpha_k|\alpha_k\rangle\right)\right]. \quad \text{(S17)}$$

Using the Fock state basis representation $|\alpha_k\rangle = e^{-|\alpha_k|^2/2}\sum_{n=0}^{\infty}\alpha_k^n (n!)^{-1/2}|n\rangle$,

$$|\partial_r\alpha_k\rangle = -\mathrm{Re}\left(\alpha_k\partial_r\alpha_k^*\right)e^{-|\alpha_k|^2/2}\sum_{n=0}^{\infty}\frac{\alpha_k^n}{(n!)^{1/2}}|n\rangle + \partial_r\alpha_k e^{-|\alpha_k|^2/2}\sum_{n=0}^{\infty}\frac{n\alpha_k^{n-1}}{(n!)^{1/2}}|n\rangle. \quad \text{(S18)}$$

Using $\sum_{n=0}^{\infty}|\alpha_k|^{2n}(n!)^{-1} = e^{|\alpha_k|^2}$, $\sum_{n=0}^{\infty}(n+1)|\alpha_k|^{2n}(n!)^{-1} = \left(1+|\alpha_k|^2\right)e^{|\alpha_k|^2}$, and the orthonormality of Fock states,

$$\langle\partial_p\alpha_k|\partial_q\alpha_k\rangle = \mathrm{Re}(\mathcal{P})\mathrm{Re}(\mathcal{Q}) - \mathrm{Re}(\mathcal{P})\mathcal{Q}^* - \mathrm{Re}(\mathcal{Q})\mathcal{P} + \partial_p\alpha_k^*\partial_q\alpha_k\left(1+|\alpha_k|^2\right) \quad \text{(S19)}$$

where $\mathcal{P} = \alpha_k\partial_p\alpha_k^*$ and $\mathcal{Q} = \alpha_k\partial_q\alpha_k^*$. Equation (S19) can be further reduced to

$$\left\langle \partial_p \alpha_k \middle| \partial_q \alpha_k \right\rangle = \partial_p \alpha_k^* \partial_q \alpha_k + \mathrm{Im}(\mathcal{P})\,\mathrm{Im}(\mathcal{Q}). \tag{S20}$$

Meanwhile, using $\left|\alpha_k\right\rangle = e^{-|\alpha_k|^2/2} \sum_{n=0}^{\infty} \alpha_k^n (n!)^{-1/2} \left|n\right\rangle$, $\sum_{n=0}^{\infty} |\alpha_k|^{2n} (n!)^{-1} = e^{|\alpha_k|^2}$, and the orthonormality of Fock states, we can obtain $\left\langle \alpha_k \middle| \partial_p \alpha_k \right\rangle = \mathrm{Im}\,\mathcal{P}$ and $\left\langle \partial_q \alpha_k \middle| \alpha_k \right\rangle = \mathrm{Im}\,\mathcal{Q}$ from Eq. (S18). Therefore, from Eq. (S17),

$$\mathcal{I}_{pq} = 4 \sum_{k=1}^{N} \mathrm{Re}\left(\partial_p \alpha_k^* \partial_q \alpha_k \right). \tag{S21}$$

We now specialize to the estimation of Slepian–Pollak coefficients of a confined object source profile $\sum_i c_i S_i(x)$ using measurement of its radiated field $\sum_i \gamma_i c_i S_i(x')$ (Fig. 1b in the main text). By choosing $\left|\alpha_k\right\rangle$ as the state vector representing kth single Slepian-Pollak mode, we can obtain $\partial_p \alpha_k = \beta \gamma_p \delta_{pk}$ where δ and β are the Kronecker delta and the normalization constant, respectively. Therefore, from Eq. (S21),

$$\mathcal{I}_{pq} = 4 \left|\beta \gamma_p\right|^2 \delta_{pq}\,. \tag{S22}$$

Because the quantum Cramér–Rao bound for ith parameter estimation for a simultaneous N parameters estimation $\sigma^2_{\mathrm{QCRB},i}$ can be represented as $\sigma^2_{\mathrm{QCRB},i} = (\mathcal{I}^{-1})_{ii}$, Eq. (S22) yields

$$\frac{\sigma^2_{\mathrm{QCRB},1}}{\sigma^2_{\mathrm{QCRB},i}} = \left|\frac{\gamma_i}{\gamma_1}\right|^2. \tag{S23}$$

Equation (S23) is the key relation used to derive the fundamental performance bound in the LSOM (graphically illustrated in Supplementary Fig. S1).

Next, to relate this to resolution, we follow *number of degrees of freedom* arguments described in main text Ref. 19, 22: for a field confined to a region of size D, the effective spatial resolution can be roughly quantified as D/M where M is the number of Slepian–Pollak coefficients that can be reliably estimated. The signal-to-noise ratio of the ith coefficient SNR_i becomes

$$\mathrm{SNR}_i = \mathrm{SNR}_1 \frac{\sigma^2_{\mathrm{QCRB},1}}{\sigma^2_{\mathrm{QCRB},i}} = \mathrm{SNR}_1 \left|\frac{\gamma_i}{\gamma_1}\right|^2 \tag{S24}$$

assuming scattered photons are mostly from the 1st Slepian-Pollak mode, $\mathrm{SNR}_1 \approx P_{\mathrm{tot}}$ following Poisson statistics where P_{tot} is the total number of scattered photons. Assuming $\gamma_1 \approx 1$, Eq. (S24) yields

$$\mathrm{SNR}_i = P_{\mathrm{tot}} \left|\gamma_i\right|^2 .$$

(S25)

Because $|\gamma_i|$ monotonically decreases with i, there exists a maximum index M such that $\mathrm{SNR}_M \approx P_{\mathrm{tot}}|\gamma_M|^2 \sim 1$, leads directly to Eq. (1) in the main text and to the scaling relationships between resolution, photon budget, and field-of-view size.

3. Classical Cramér–Rao bound and signal-to-noise ratio for our measurement scheme.

We first derive the classical Fisher information matrix, $\mathcal{J}(c_1,...,c_N)$ and the corresponding Cramér–Rao bounds for simultaneous estimation of N Slepian–Pollak coefficients using our N'-channel measurement scheme ($N' \geq N$), following Supplementary Ref. 7 and main text Ref. 38.

For a parameter vector $\boldsymbol{\theta}$ and a random data vector $\mathbf{X}$, the (p, q) element of the Fisher information matrix is defined as[7]

$$\mathcal{J}_{pq} = -\mathbb{E}\left[\frac{\partial^2 \ln p(\mathbf{X};\boldsymbol{\theta})}{\partial\theta_p \partial\theta_q}\right] \tag{S26}$$

where $\mathbb{E}[A]$ is the expectation value of A and $p(\mathbf{X}; \boldsymbol{\theta})$ is a joint probability density function of $\mathbf{X}$, respectively. In our case, $\mathbf{X}$ is the N'-by-1 complex random vector of measured coefficients X_k (k = 1, …, N') obtained from the eight-step interferometric scheme described in Supplementary Methods 2. From Eq. (S1), we can write

$$X_k = \frac{1}{4}\sum_{v=1}^{4}\left[\frac{i^v X_{\mathrm{a},v}^{(k)}}{\sqrt{I_\mathrm{a}}} + e^{i\phi_{\mathrm{ba}}}\frac{i^v X_{\mathrm{b},v}^{(k)}}{\sqrt{I_\mathrm{b}}}\right] \tag{S27}$$

where $X_{u,v}^{(k)}$ (u = a, b) are the random variables of measured raw data—i.e. photon number per frame (= per repetition) at a pixel with the respective DMD mask—with the expectation value of $I_{u,v}^{(k)}$, and $I_{u,v}^{(k)} = \left|E_u^{(\mathrm{ref})} + (-i)^v E_u^{(k)}\right|^2$. Because $E_u^{(\mathrm{ref})}$ is strong enough for our measurement, we can assume a normal distribution for $X_{u,v}^{(k)}$ where the variance is the shot noise of the reference mode, I_u [8]. Because $X_{u,v}^{(k)}$ are independent for different u and v, and assuming $\angle\left[E_\mathrm{a}^{(\mathrm{ref})}\right] = 0$, the random variable $X_\mathrm{a}^{(k)}\left(=\sum_{v=1}^{4} i^v X_{\mathrm{a},v}^{(k)}/\sqrt{I_\mathrm{a}}\right)$ follows a complex normal distribution with a variance of 4 and an expectation value of $4E_\mathrm{a}^{(k)}$ where $\left|E_\mathrm{a}^{(k)}\right|^2$ is the expectation value of the photon number (per pixel per frame) that will be measured if s_k^* is realized at the right-half of the DMD mask without anything at the left-half (Supplementary Fig. S13). Similarly, assuming $\angle\left[e^{i\phi_{\mathrm{ba}}} E_\mathrm{b}^{(\mathrm{ref})}\right] = 0$, $X_\mathrm{b}^{(k)}\left(= e^{i\phi_{\mathrm{ba}}}\sum_{v=1}^{4} i^v X_{\mathrm{b},v}^{(k)}/\sqrt{I_\mathrm{b}}\right)$ follows a complex normal distribution with a variance of 4 and an expectation value of $4E_\mathrm{b}^{(k)}$ where $\left|E_\mathrm{b}^{(k)}\right|^2$ is the expectation value of the photon number (per pixel per frame) that will be measured if s_k^* is realized at the left-half of the DMD mask without anything at the right-half (Supplementary Fig. S13). Therefore, $X_k\left(= X_\mathrm{a}^{(k)} + X_\mathrm{b}^{(k)}\right)$ follows a complex normal distribution

with a variance of 2 and an expectation value of $E^{(k)}$ $(=E_{\mathrm{a}}^{(k)}+E_{\mathrm{b}}^{(k)})$ where $\left|E^{(k)}\right|^2$ is the expectation value of the photon number (per pixel per frame) that will be measured if s_k^* is realized at the DMD mask (Supplementary Fig. S13). Then, the joint probability distribution for a random vector $\mathbf{X}$ becomes

$$p(\mathbf{X},\boldsymbol{\theta})=\prod_{k=1}^{M}\frac{1}{2\pi}\exp\left[-\frac{\left|X_k-E^{(k)}\right|^2}{2}\right]. \tag{S28}$$

For R_{kk} repetitions of the kth coefficient measurement (for all 8 sub-measurements), the effective variance of the averated X_k is reduced by R_{kk}, and the joint probability distribution becomes

$$p_{\mathrm{rep}}(\mathbf{X},\boldsymbol{\theta})=\prod_{k=1}^{M}\frac{1}{2\pi}\exp\left[-\frac{\left|X_k-E^{(k)}\right|^2}{2R_{kk}}\right]. \tag{S29}$$

Substituting Eq. (S28) into Eq. (S26), we obtain for a single measurement

$$\begin{aligned}\mathcal{J}_{pq}&=\frac{1}{2}\mathbb{E}\left[\sum_{k=1}^{M}\frac{\partial^2\left[\left(X_k-E^{(k)}\right)\left(X_k^*-E^{(k)*}\right)\right]}{\partial\theta_p\partial\theta_q}\right]\\&=\frac{1}{2}\mathbb{E}\left[\sum_{k=1}^{M}\partial_q\left[-\partial_pE^{(k)}\left(X_k^*-E^{(k)*}\right)-\left(X_k-E^{(k)}\right)\partial_pE^{(k)*}\right]\right]\\&=\frac{1}{2}\mathbb{E}\left[\sum_{k=1}^{M}\left[-\partial_q\partial_pE^{(k)}\left(X_k^*-E^{(k)*}\right)+\partial_pE^{(k)}\partial_qE^{(k)*}+\right.\right.\\&\qquad\left.\left.\partial_qE^{(k)}\partial_pE^{(k)*}-\left(X_k-E^{(k)}\right)\partial_q\partial_pE^{(k)*}\right]\right]\\&=\sum_{k=1}^{M}\mathrm{Re}\left[\partial_pE^{(k)}\partial_qE^{(k)*}\right]\end{aligned} \tag{S30}$$

and for the repetitive case

$$\mathcal{J}_{\text{rep},pq} = \sum_{k=1}^{M} R_{kk} \operatorname{Re}\left[\partial_p E^{(k)} \partial_q E^{(k)*}\right]. \tag{S31}$$

We now express $E^{(k)}$ in terms of the transfer matrix. Let

$$\mathbf{E} = \beta T \mathbf{c}_{\text{o}} \tag{S32}$$

where $\mathbf{E} = \left(E^{(1)}, E^{(1)}, \dots, E^{(N')}\right)^t$, β, $\mathbf{c}_{\text{o}} = \left(c_{\text{o},1},\ c_{\text{o},2},\ \dots,\ c_{\text{o},N}\right)^t$, and T are the vector of expected values of measured coefficients, the normalization constant, the vector of the true Slepian–Pollak coefficients, and the N'-by-N transfer matrix, respectively, and t denotes the transpose. Using orthogonality of Slepian–Pollak functions, $E^{(k)} = \beta \sum_{r=1}^{N} T_{kr} c_r$, yielding $\partial_r E^{(k)} = \beta T_{kr}$. Then, the Fisher information matrix without repetition becomes $\mathcal{J}_{pq} = |\beta|^2 \sum_{k=1}^{M} \operatorname{Re}\left[T_{kq}^{*} T_{kp}\right] = |\beta|^2 \operatorname{Re}\left[\sum_{k=1}^{M} (T^H)_{qk} T_{kp}\right] = |\beta|^2 \operatorname{Re}\left[(T^H T)_{qp}\right] = |\beta|^2 \operatorname{Re}\left[(T^H T)_{pq}\right]$, yielding $\mathcal{J} = |\beta|^2 \operatorname{Re}\left[T^H T\right]$. For R_{kk} repetitions, the Fisher information matrix becomes $\mathcal{J}_{\text{rep},pq} = |\beta|^2 \operatorname{Re}\left[\sum_{k=1}^{M} (T^H)_{qk} R_{kk} T_{kp}\right] = |\beta|^2 \operatorname{Re}\left[(T^H R T)_{qp}\right] = |\beta|^2 \operatorname{Re}\left[(T^H R T)_{pq}\right]$ where R is the N'-by-N' diagonal matrix with kth diagonal elements R_{kk}, yielding $\mathcal{J}_{\text{rep}} = |\beta|^2 \operatorname{Re}\left[T^H R T\right]$. The classical Cramér–Rao bound for simultaneous estimation of $c_{\text{o,i}}$ ($i = 1, \dots, N$) with a set of repetitive measurement is $\sigma^2_{\text{CRB},R,i} = (\mathcal{J}^{-1}_{\text{rep}})_{ii}$. In terms of accuracy—reciprocal of $\sigma^2_{\text{CRB},R,i}$—normalized by the accuracy for the estimation of reference (i.e. rth) mode coefficient without repetition, we can write

$$\frac{\sigma^2_{\text{CRB},1,r}}{\sigma^2_{\text{CRB},R,i}} = \frac{(\mathcal{J}^{-1})_{rr}}{(\mathcal{J}^{-1}_{\text{rep}})_{ii}} = \frac{[\{\text{Re}(T^H T)\}^{-1}]_{rr}}{[\{\text{Re}(T^H RT)\}^{-1}]_{ii}}. \quad \text{(S33)}$$

Equation (S33) is the key result used to derive Eq. (2) in the main text: it connects the achievable accuracy in estimating $c_{\text{o},i}$ with the transfer matrix T and the repetition R.

Next, the corresponding signal-to-noise ratio for the ith coefficient with repetitions specified by R is,

$$\text{SNR}_{R,i} = \text{SNR}_{1,r} \frac{\sigma^2_{\text{CRB},1,r}}{\sigma^2_{\text{CRB},R,i}} = \text{SNR}_{1,r} \frac{[\{\text{Re}(T^H T)\}^{-1}]_{rr}}{[\{\text{Re}(T^H RT)\}^{-1}]_{ii}} \quad \text{(S34)}$$

where $\text{SNR}_{1,r}$ is the signal-to-noise ratio for the reference mode without repetition. If the detected photons arise predominantly from the reference mode, $\text{SNR}_{1,r} \approx P_{\text{sin}}$, where P_{sin} is the number of photons measured per sub-measurement at the selected pixel. The condition $\text{SNR}_{R,I} \geq 1$ yields Eq. (2) in the main text, and provides a practical bound for choosing R to ensure reliable coefficient estimation.

4. Overall point spread function.

Because $|\gamma_i/\gamma_1|^2$ rapidly decreases with i, the source amplitude for a TIR-illuminated object can be well approximated by a truncated Slepian–Pollak series $\mathbf{c}_\text{o} = F\mathbf{a}_\text{o}$ where $\mathbf{c}_\text{o}$ is the N-by-1 vector of true Slepian–Pollak coefficients, F is an N-by-P generalized Fourier transform matrix that maps a P-point spatial representation to the truncated Slepian–Pollak basis, and $\mathbf{a}_\text{o}$ is the P-by-1 vector of source amplitudes in the position basis (discretized x in main text Fig. 1b in the main text).

For four illumination directions (2D imaging), we define the concatenated coefficient vectors $\mathbf{c}'_\text{o} = \left(\mathbf{c}^t_{\text{o},1}, \mathbf{c}^t_{\text{o},2}, \mathbf{c}^t_{\text{o},3}, \mathbf{c}^t_{\text{o},4}\right)^t$, $\mathbf{a}'_\text{o} = \left(\mathbf{a}^t_{\text{o},1}, \mathbf{a}^t_{\text{o},2}, \mathbf{a}^t_{\text{o},3}, \mathbf{a}^t_{\text{o},4}\right)^t$, and generalized transform $F' = \text{diag}\left(F, F, F, F\right)$, so that $\mathbf{c}'_\text{o} = F'\mathbf{a}'_\text{o}$ where $\mathbf{a}_{\text{o},l}$ and $\mathbf{c}_{\text{o},l}$ denote the contributions for the

lth illumination. The stacked measurement vector of Slepian–Pollak coefficients is $\mathbf{c}'_{\mathrm{m}} = T'\mathbf{c}'_{\mathrm{o}}$ where $T' = \mathrm{diag}(T,T,T,T)$ ($N' = N$ as in our experiment). The vector linear minimum mean square error estimator (Supplementary Methods 4) for the full coefficient vector is $W' = \mathrm{diag}(W,W,W,W)$, so that $\mathbf{c}'_{\mathrm{e}} = W'\mathbf{c}'_{\mathrm{m}}$. The estimated source amplitude in the position basis is then $\mathbf{a}'_{\mathrm{e}} = F'_{\mathrm{inv}}\mathbf{c}'_{\mathrm{e}}$ where $F'_{\mathrm{inv}} = \mathrm{diag}(F_{\mathrm{inv}}, F_{\mathrm{inv}}, F_{\mathrm{inv}}, F_{\mathrm{inv}})$ and F_{inv} is the generalized inverse Fourier transform matrix that maps the truncated Slepian–Pollak representation back to the position basis. The final reconstructed object amplitude (e.g. polarizability) is the coherent sum of the contributions from each illumination, including phase corrections, $\mathbf{a}_{\mathrm{r}} = V'\mathbf{a}'_{\mathrm{e}}$ where $V' = (V_1, V_2, V_3, V_4)$, and V_l is a diagonal matrix encoding position-dependent phase correction factors derived from the TIR illumination wavevectors $V_l(\mathbf{r}) = e^{-i(\mathbf{k}_{\mathrm{TIR},l}\cdot\mathbf{r}+\phi_l)}$ and global phase corrections $\phi_l = \angle\left[\int_{|\mathbf{r}|\le R_{\mathrm{O}}}\left(\sum_{k=1}^{N}\mathbf{c}_{\mathrm{e},l}(k)S_k(\mathbf{r})\right)d\mathbf{r}\right]$ (Supplementary Methods 3). Combining these relations, the reconstruction process can be written as a linear mapping,

$$\mathbf{a}_{\mathrm{r}} = \left[V'F'_{\mathrm{inv}}W'T'F'\right]\mathbf{a}'_{\mathrm{o}}. \tag{S35}$$

Equation (S35) implies that our imaging process is a coherent linear imaging because the amplitudes are in a linear relationship (whereas intensities are in the linear relationship in incoherent imaging). The amplitude point spread function (PSF) becomes,

$$\mathbf{p}_{\mathrm{r}} = \left[V'F'_{\mathrm{inv}}W'T'F'\right]\mathbf{p}'_{\mathrm{o}} \tag{S36}$$

where $\mathbf{p}'_{\mathrm{o}} = \left(\mathbf{p}^t_{\mathrm{o}}, \mathbf{p}^t_{\mathrm{o}}, \mathbf{p}^t_{\mathrm{o}}, \mathbf{p}^t_{\mathrm{o}}\right)^t$ and $\mathbf{p}_{\mathrm{o}}$ is a point source. For a single illumination without using V_l and an ideal filter ($W = T^{-1}$), Eq. (S36) reduces to $\mathbf{p}_{\mathrm{r}} = \left[F_{\mathrm{inv}}F\right]\mathbf{p}_{\mathrm{o}}$, equivalent to amplitude PSF described in the main text Ref. 12. As in a conventional coherent linear imaging, the amplitude PSF is a suitable measure for the performance of LSOM because Eq. (S35) with arbitrary $\mathbf{a}'_{\mathrm{o}}$ can be constructed with a superposition of Eq. (S36). The PSF is slightly spatially variant reflecting quasi-uniform nature of the Slepian–Pollak basis[9]. Supplementary Fig. S5

shows both calculated and measured PSFs, mapped to Cartesian coordinates and evaluated for several impulse positions.

Supplementary Figures

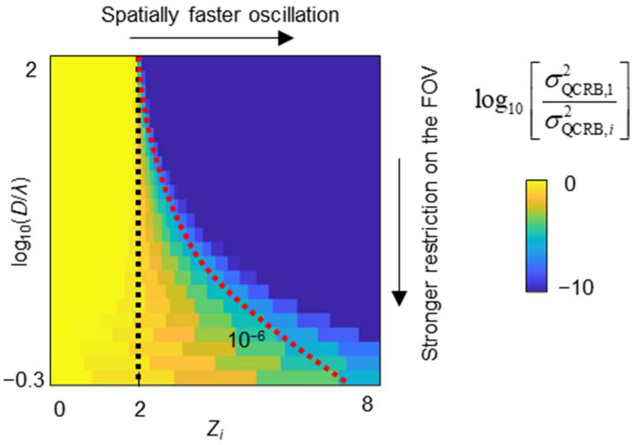

Figure S1. Maximum attainable accuracy for Slepian–Pollak coefficient estimation. Quantum Cramér–Rao bound (Eq. (S23)) for estimating Slepian–Pollak coefficients as a function of field-of-view (FOV) size. The normalized estimation accuracy depends on both the average number of zeros per wavelength Z_i (horizontal axis) of the ith Slepian–Pollak function within the FOV and on D/λ (vertical axis), where D is the physical FOV size. Z_i characterizes the oscillation rate of the ith Slepian–Pollak function within the FOV.

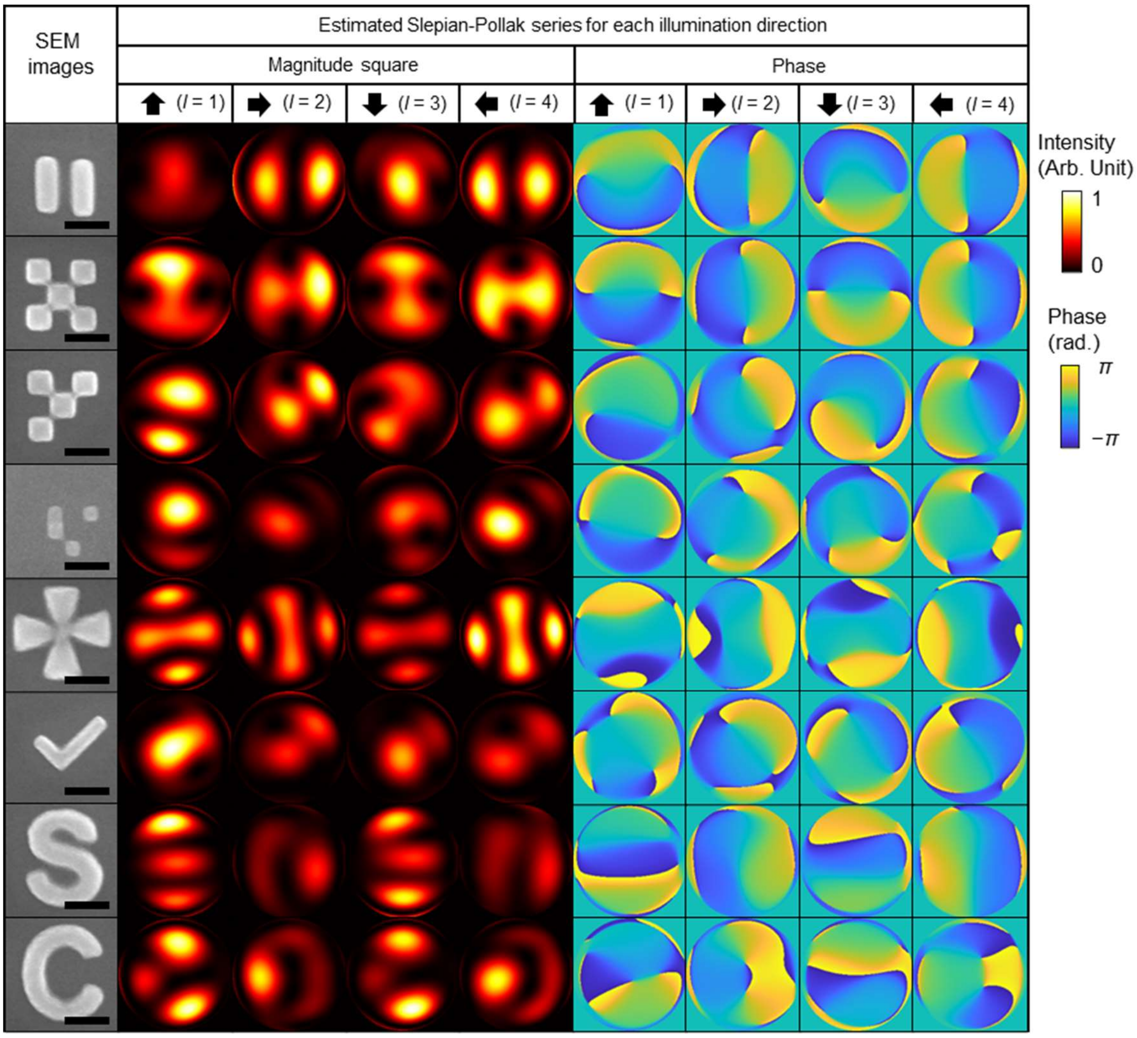

Figure S2. Reconstructed limited-size object microscopy (LSOM) images for each object and illumination direction (2D imaging). Arrows indicate the in-plane component of the illumination wavevector. Scale bar in SEM images: 200 nm.

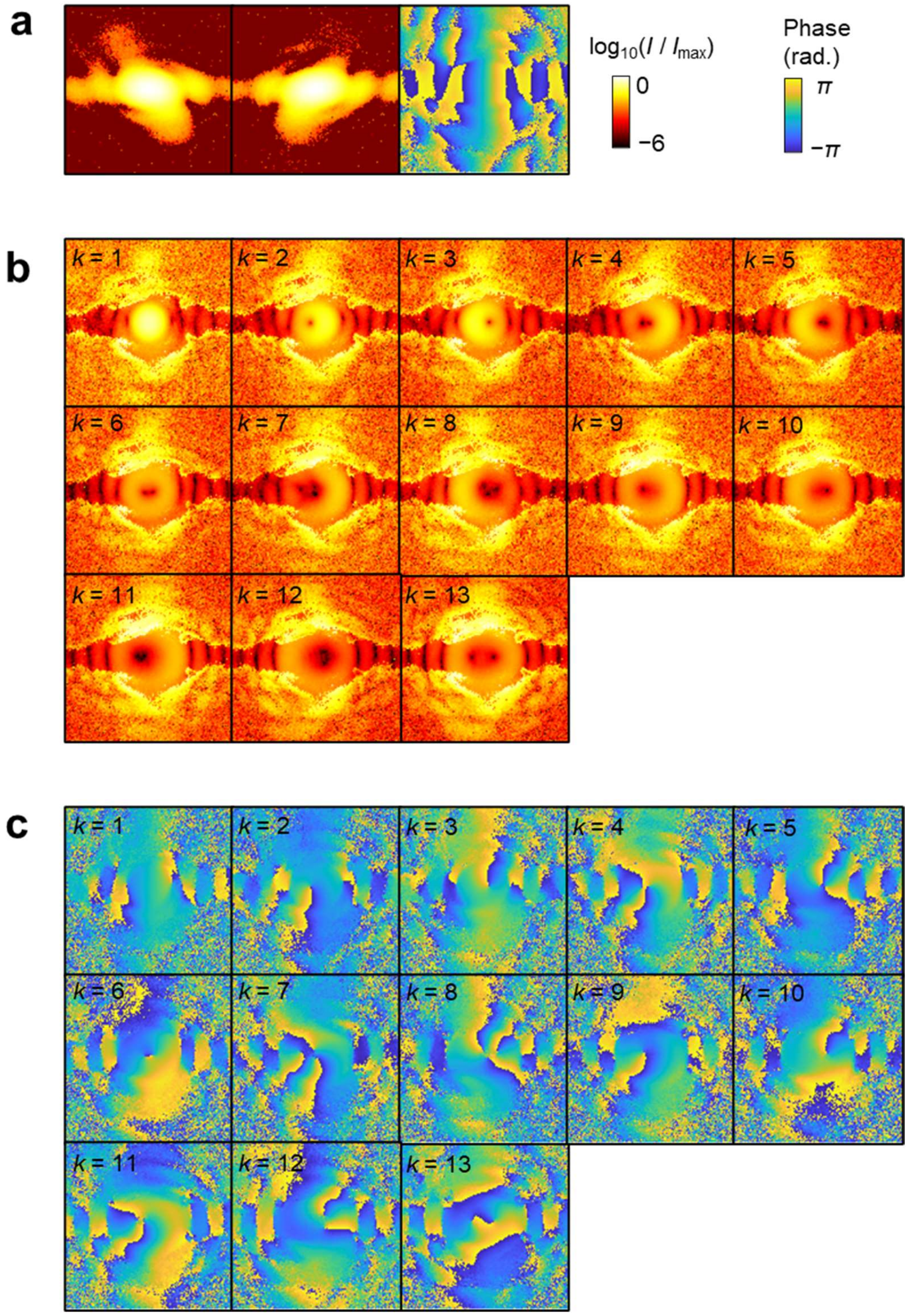

Figure S3. Example raw data from the camera for 2D limited-size object microscopy (LSOM). Representative dataset for object #1 in Supplementary Fig. S2 (two bars) and illumination direction $l = 1$. (a) Measured I_a, I_b, and ϕ_{ba}, from left to right. (b) Modulus of one realization of X_k (Eq. (S27)) for all k. (c) Phase of the same realization of X_k.

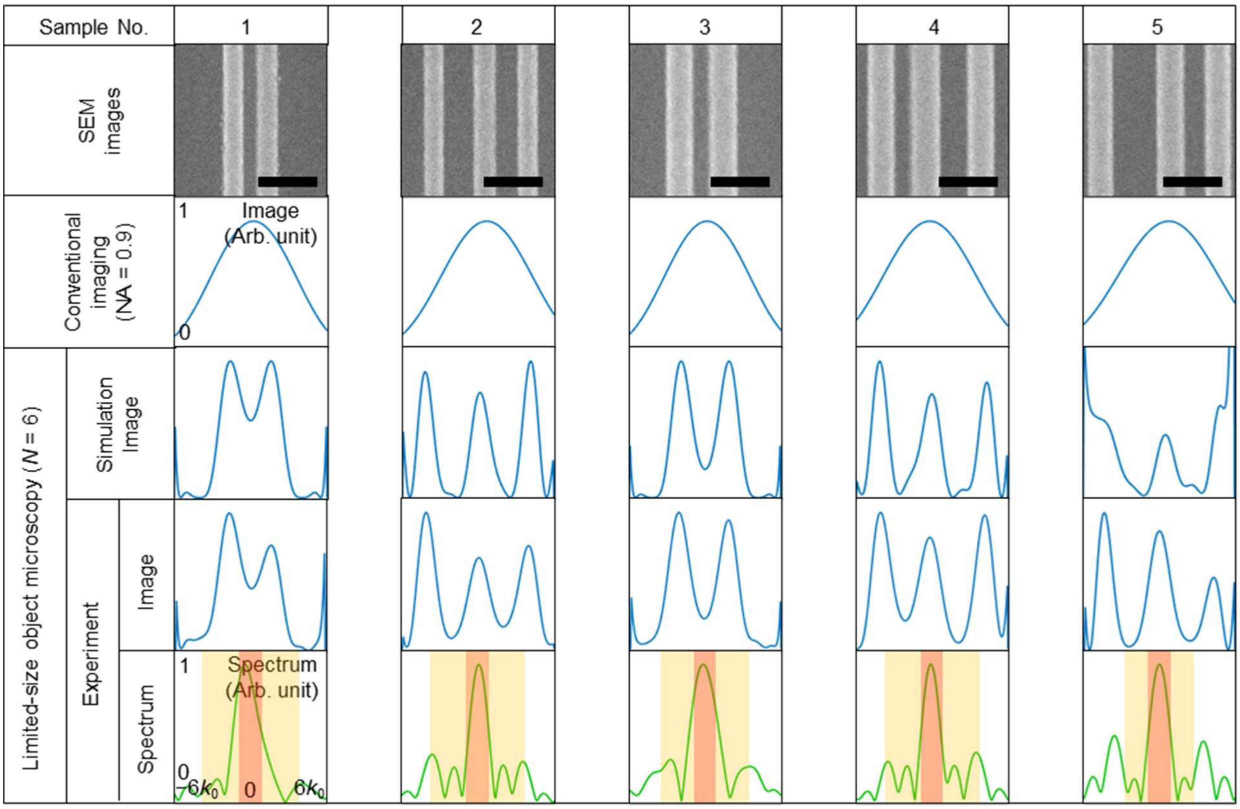

Figure S4. Experimental demonstration of 1D limited-size object microscopy (LSOM). Scanning electron microscope images of nanoscale line arrays with different numbers, widths, and gaps (top row, scale bar: 200 nm) are compared with ideal coherent Fourier imaging with numerical aperture (NA) = 0.9 (second row), simulated (LSOM) images (third row) and experimental LSOM images (fourth row). LSOM clearly outperforms conventional imaging. The spectra of the experimental images (fifth row) reveal an effective numerical aperture of 3.53.

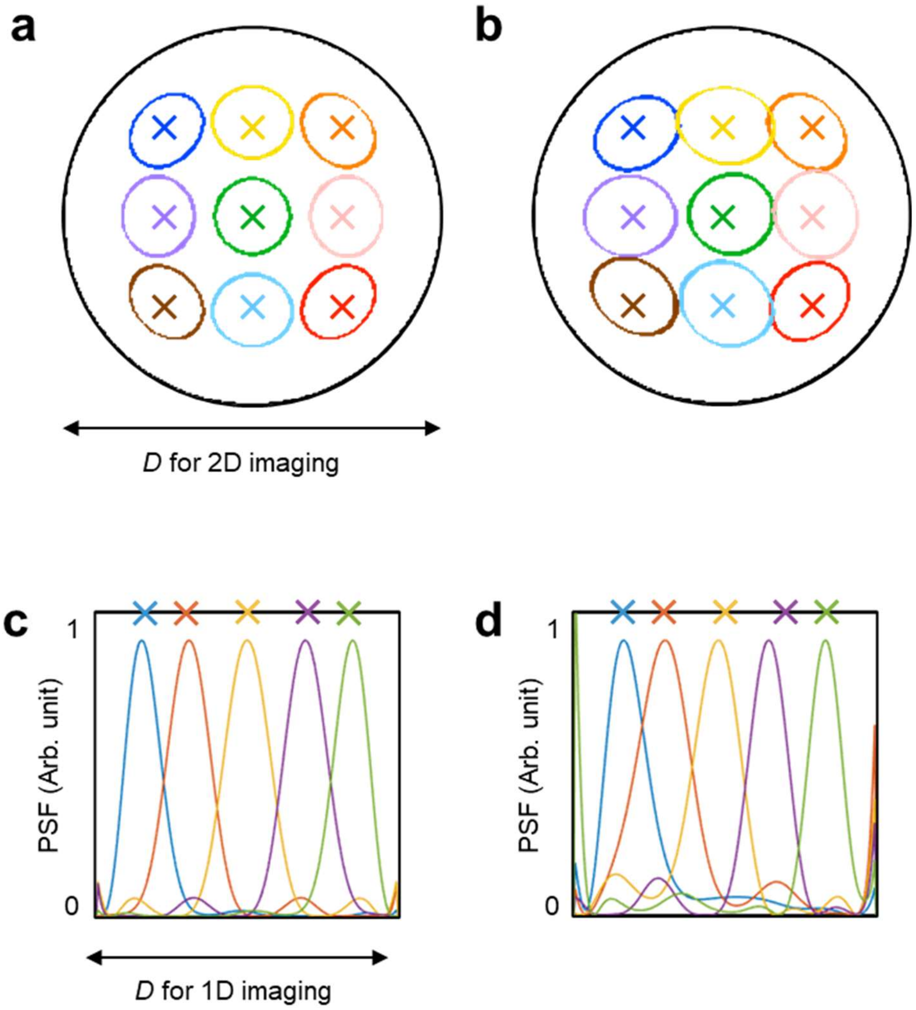

Figure S5. Overall point spread function (PSF) of limited-size object microscopy (LSOM) for different point source positions. Each point source position (marked by ×) and its corresponding PSF are shown in the same color. (a, b) 2D LSOM (contours mark the half-maximum). (c, d) 1D LSOM. (a, c) Calculated ideal overall PSF, i.e. $|\mathbf{p}_\mathrm{r}|^2 = |[V'F'_\mathrm{inv}F']\mathbf{p}'_\mathrm{o}|^2$. (b, d) Measured PSF.

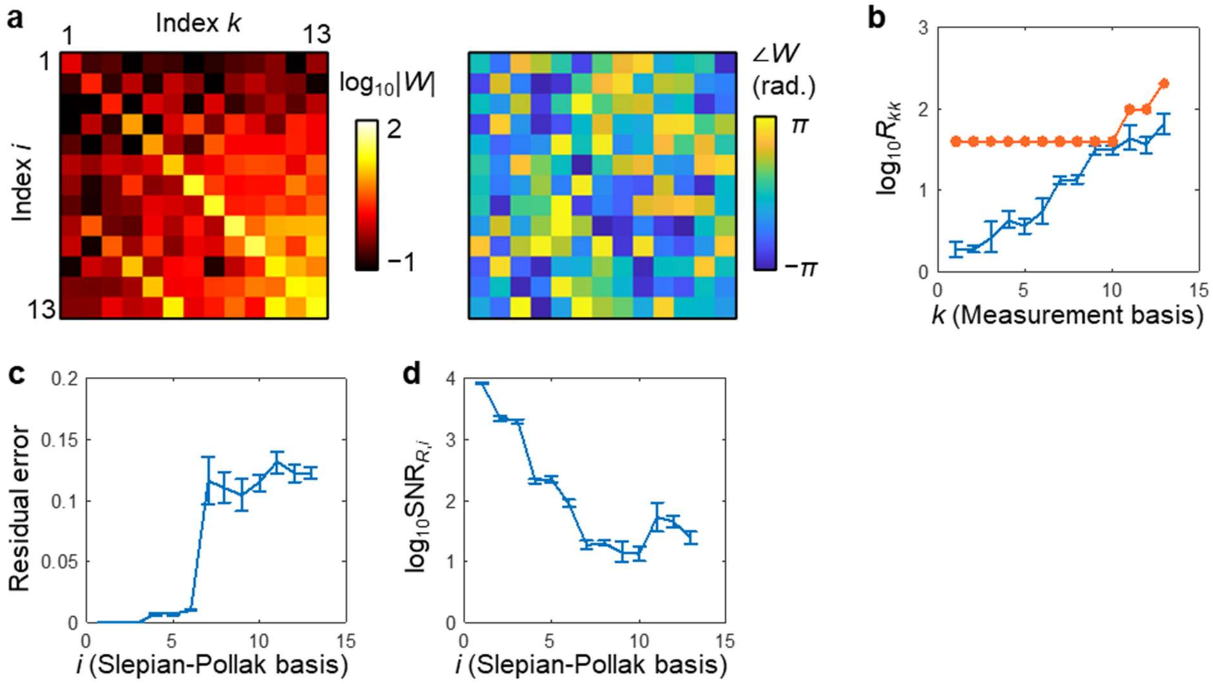

Figure S6. Characterization of the optical setup and filter performance. (a) Experimentally constructed vector linear minimum mean square error filter matrix W. (b) Required repetitions R_{kk} from Eq. (S5) (blue) and implemented repetitions (orange) for each coefficient measurement. We set $\varepsilon = 0.1$. (c) Average residual error between filtered coefficients and the true coefficients of the calibration dot, averaged over L pixels. (d) Expected signal-to-noise ratio for each coefficient, computed from Eq. (S34). Data are presented as mean values ± standard deviation ($n = 4$).

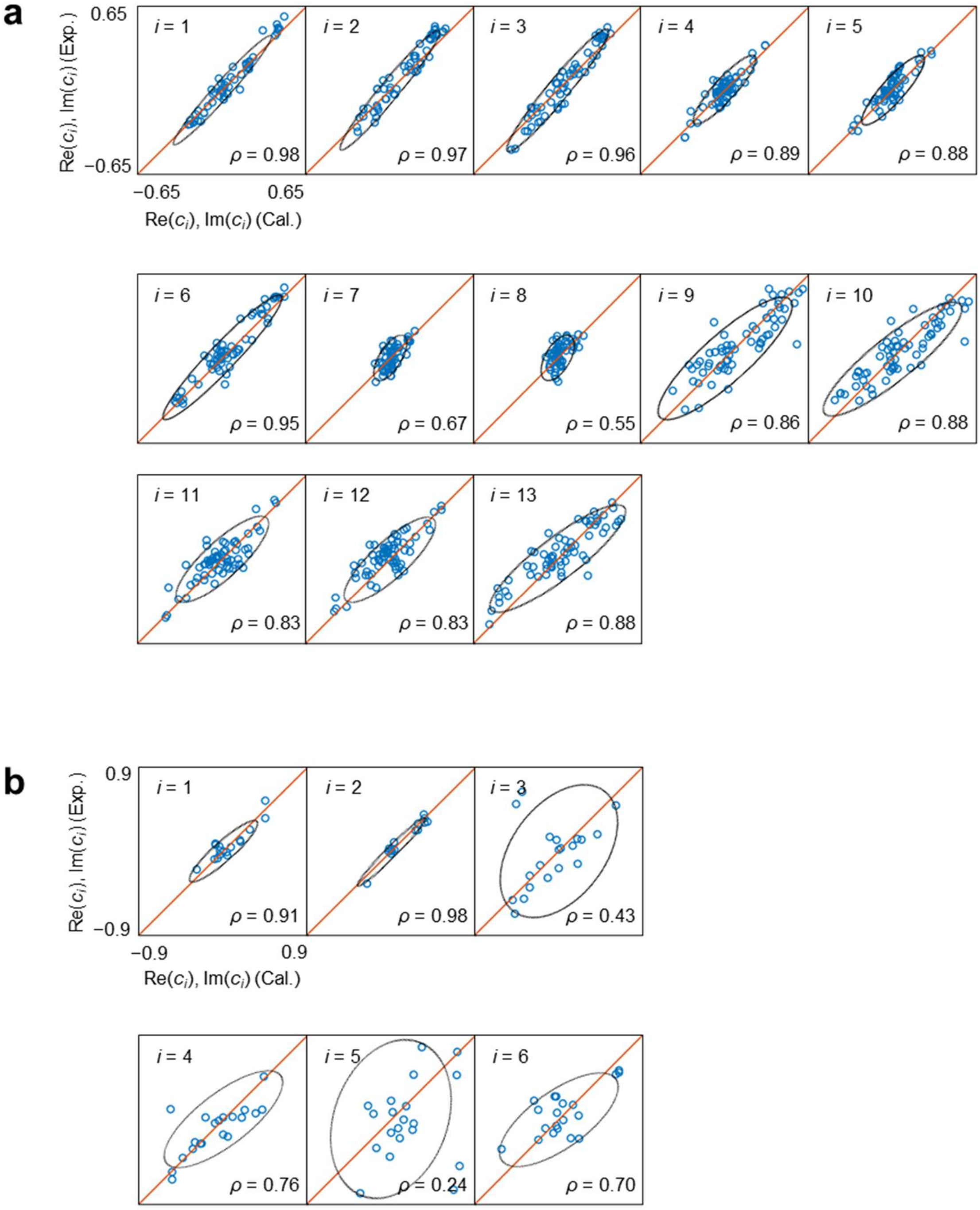

Figure S7. Comparison of experimental and simulated Slepian–Pollak coefficients. Scatter plots of real and imaginary parts of Slepian-Pollak coefficients obtained from experiment (vertical axis) versus simulation (horizontal axis), for each mode *i*. (a) 2D imaging. (b) 1D imaging. Each panel shows the Pearson correlation coefficient ρ and the 2σ covariance ellipse.

a

Index k	Corresponding indices N and n of the $R_{N,n} \cdot \exp(iN\theta)$ from Ref. 2
1	(N,n) = (0,0)
2	(1,0)
3	(−1,0)
4	(2,0)
5	(−2,0)
6	(0,1)
7	(3,0)
8	(− 3,0)
9	(1,1)
10	(−1,1)
11	(2,1)
12	(−2,1)
13	(0,2)
14	(4,0)
15	(−4,0)
16	(5,0)
17	(−5,0)

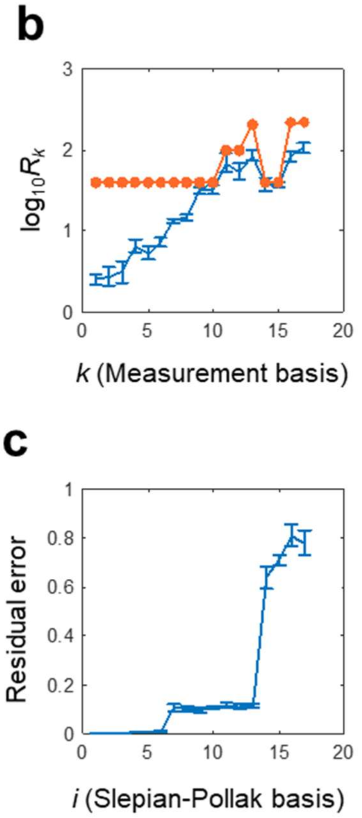

Figure S8. Degraded filter performance when using 17 coefficients (2D imaging). (a) Index k for 17 Slepian–Pollak modes, aligned with the notation in main text Ref. 30. Modes 1–13 match those of Supplementary Fig. S13a; four additional modes are appended. (b) Required repetitions (blue) and implemented repetitions (orange) for 17 coefficients; the required values are satisfied. (c) Average residual error between filtered coefficients and the true coefficients for the calibration dot. The additional modes exhibit significant residual error, despite meeting the repetition requirement. Data are presented as mean values ± standard deviation (n = 4).

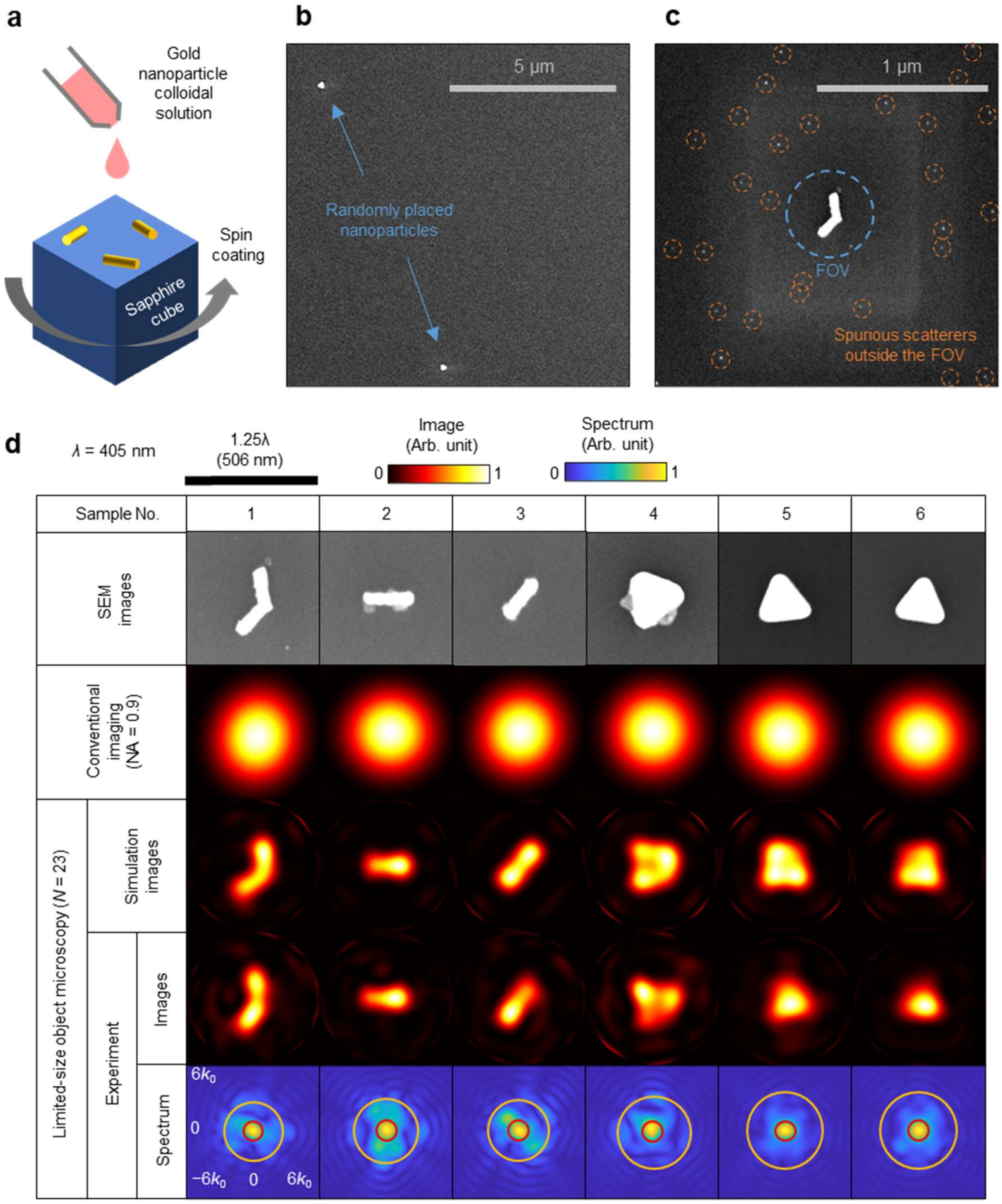

Figure S9. Limited-size object microscopy (LSOM) of randomly distributed gold nanoparticles. (a) Schematic of the sample preparation: colloidal Au nanoparticles are spin-coated onto a sapphire cube. (b) SEM image showing random distribution of nanoparticles. (c) Example where unspecified particles lie outside the field of view (object #1 in panel (d)). (d) LSOM of various Au nanoparticles, showing robust super-resolution and spectral extrapolation, while ideal conventional coherent Fourier imaging with numerical aperture (NA) = 0.9 fails to resolve the particles. λ = 405 nm is used for both the Fourier imaging and LSOM.

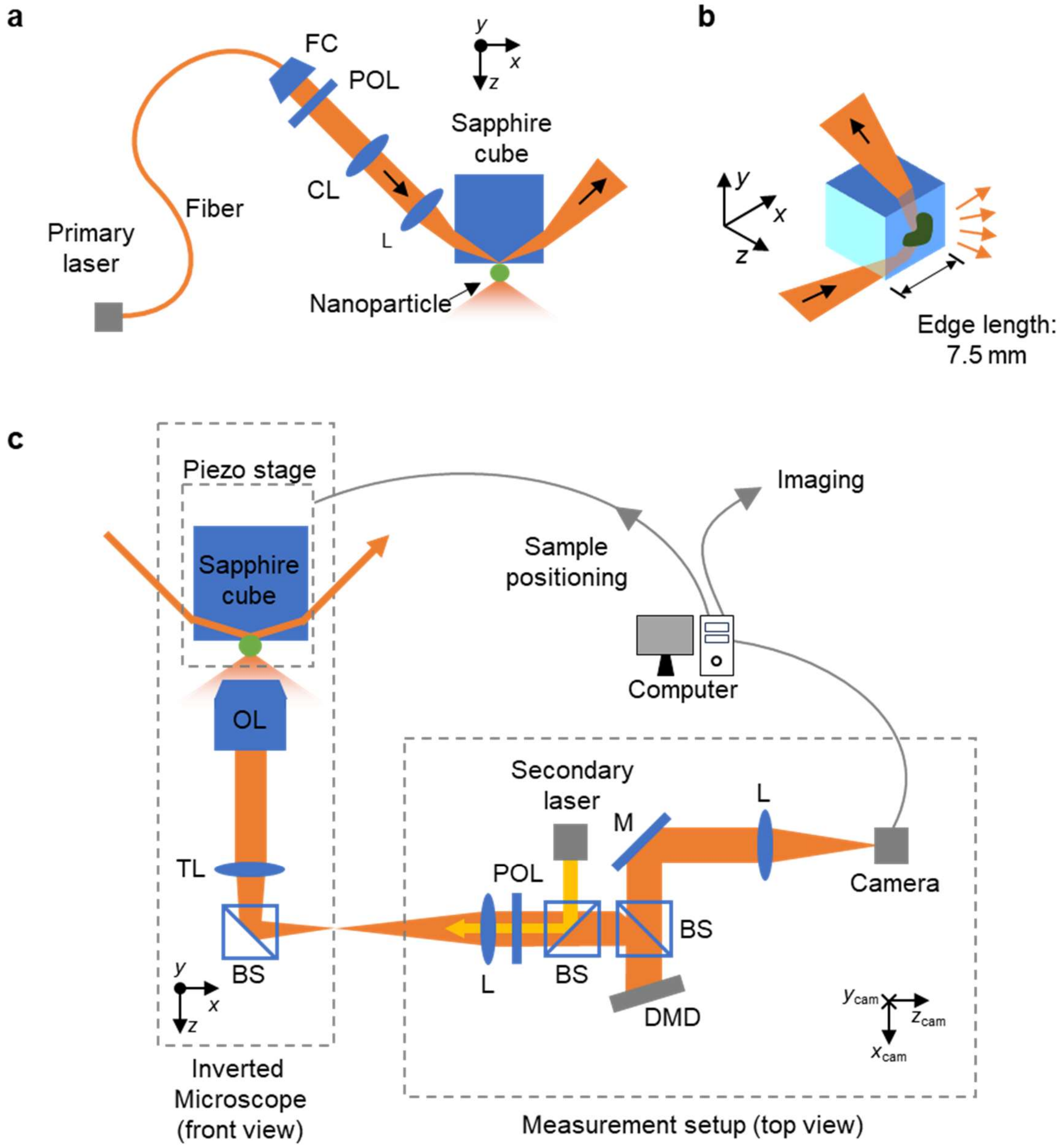

Figure S10. Optical setup (not to scale). (a) Total internal reflection (TIR) illumination realized with a sapphire cube. A fiber collimator (FC) produces a ~1 mm beam waist. A polarizer (POL) selects s-polarization. A cylindrical lens (CL) with a focal length of 500 mm pre-compensates aberrations inside the cube. A lens (L) with 50 mm focal length focuses the beam onto the object. (b) Isometric view of the sapphire cube under TIR illumination. (c) Full optical setup: the sapphire cube with nanoparticle is mounted on an inverted microscope. Scattered light is analyzed using sequential measurements with different masks. OL: objective lens, TL: tube lens, BS: beam splitter, DMD: digital micromirror device, M: mirror.

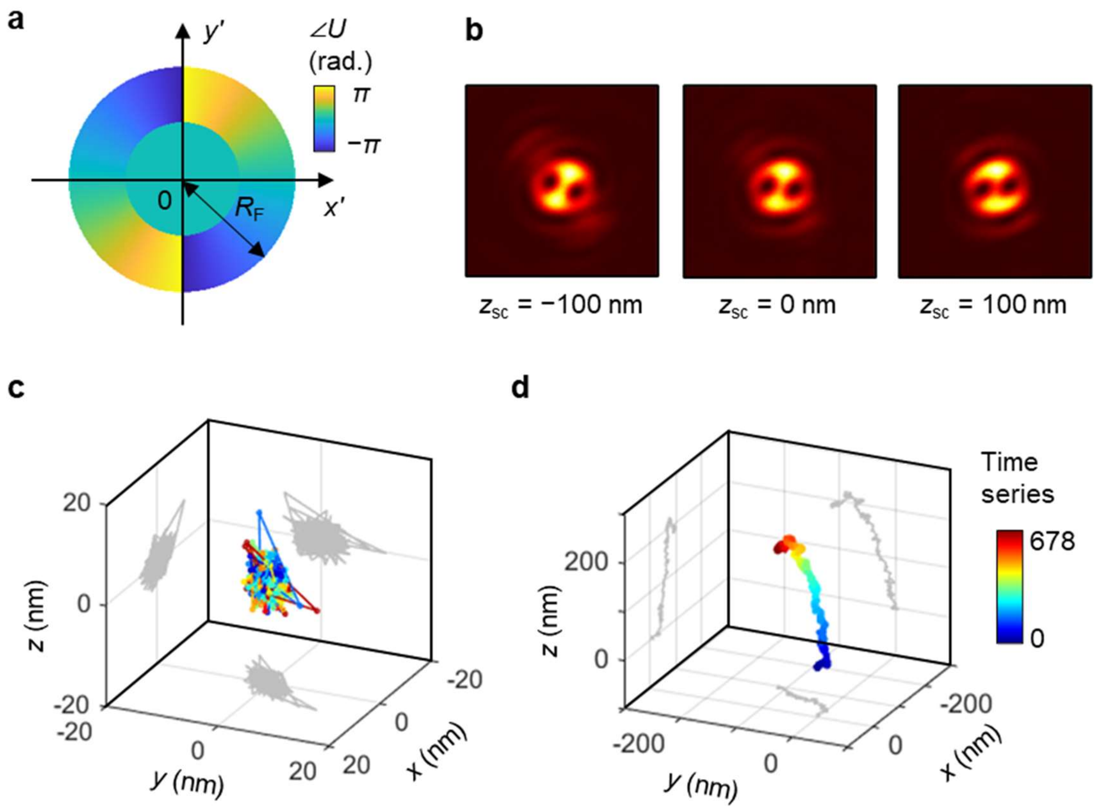

Figure S11. Real-time drift correction. (a) Phase-only mask used for 3D position estimation (uniform magnitude across the mask). (b) Example of a modified image under this mask. The pronounced *z*-dependent profile change enables *z*-localization. (c) Example of 3D drift correction during a complete measurement; standard deviations in all three coordinates are ≤ 3 nm. (d) Total 3D drift corrected by the piezo stage, inferred from applied voltage.

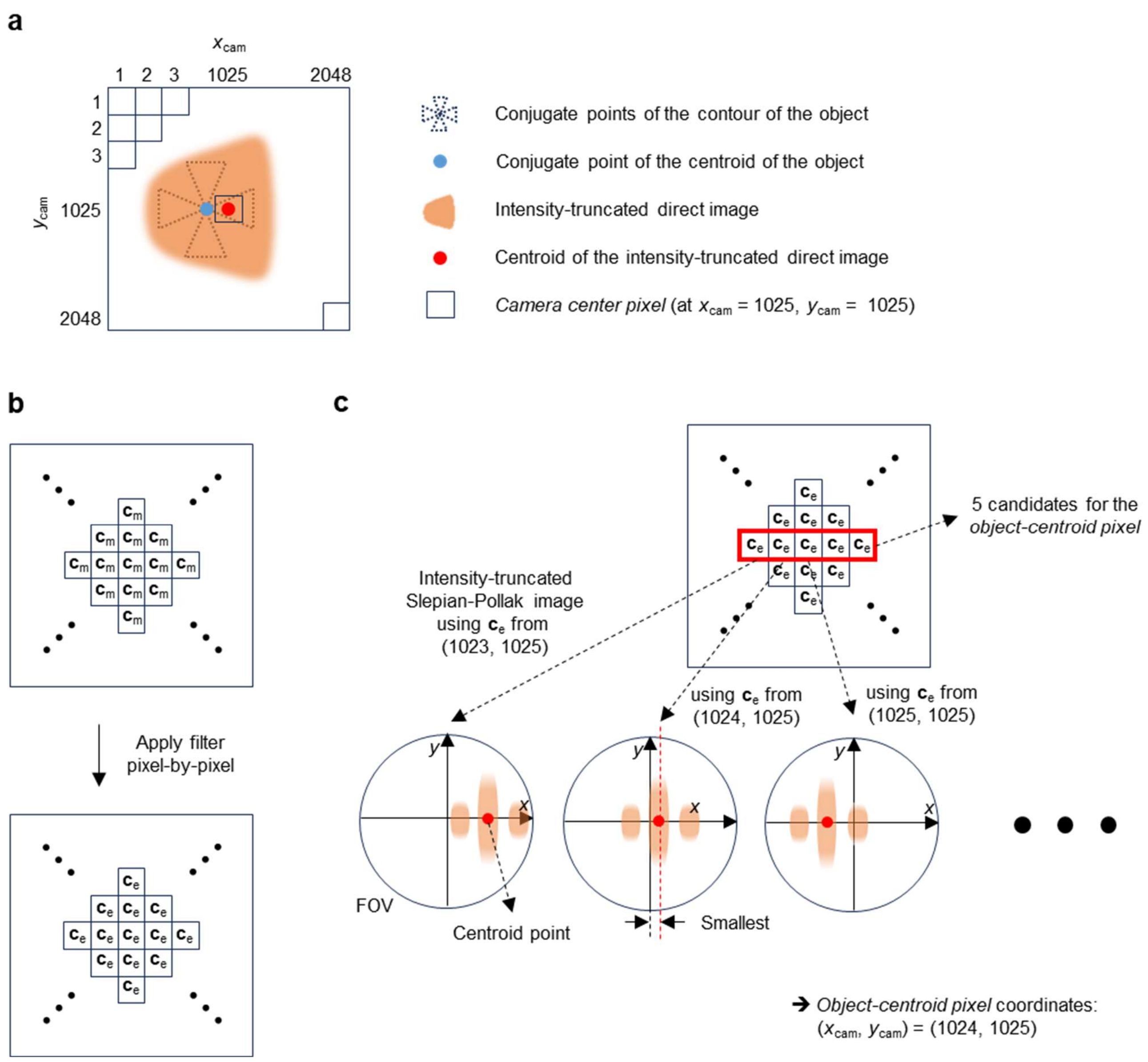

Figure S12. Camera sensor array and selection of object-centroid pixel. (a) Schematic of the camera sensor and intensity-truncated direct image (illumination along x). During object positioning, the centroid of this image is aligned to the *camera center pixel*. Because of oblique illumination, this centroid does not generally coincide with the conjugate point of the true object centroid along the illumination direction. (b) Coefficient measurement yields a spatial map of c_m; applying the filter matrix W pixel-by-pixel yields a map of c_e. Each pixel has its own coefficient vectors (indices omitted for simplicity). (c) Procedure for selecting the *object-centroid pixel* (illumination along x). LSOM images are reconstructed for five pixels (red box) around the *camera center pixel*. The pixel whose intensity-truncated LSOM image has a centroid closest to $x = 0$ is chosen as the *object-centroid pixel*. An analogous procedure is used for illumination along y.

a

Index k of the S_k in the main text	Corresponding indices N and n of the $R_{N,n}\cdot\exp(iN\theta)$ from Ref. 2
1	(N,n) = (0,0)
2	(1,0)
3	(−1,0)
4	(2,0)
5	(−2,0)
6	(0,1)
7	(3,0)
8	(− 3,0)
9	(1,1)
10	(−1,1)
11	(2,1)
12	(−2,1)
13	(0,2)

b

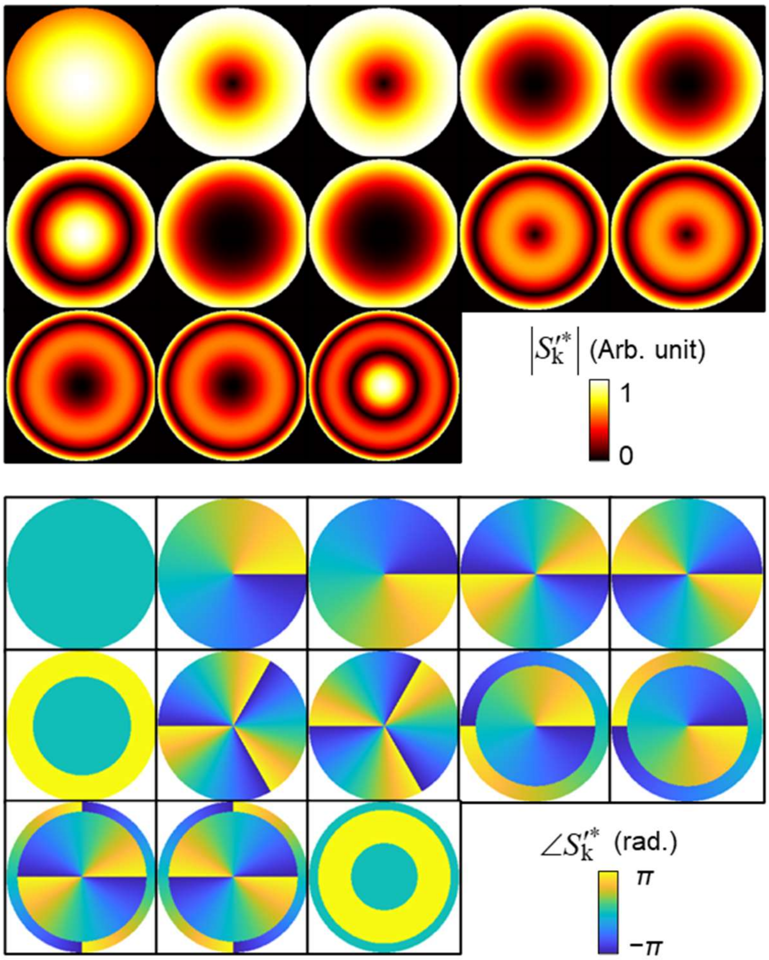

c

DMD mask pattern	Schematic	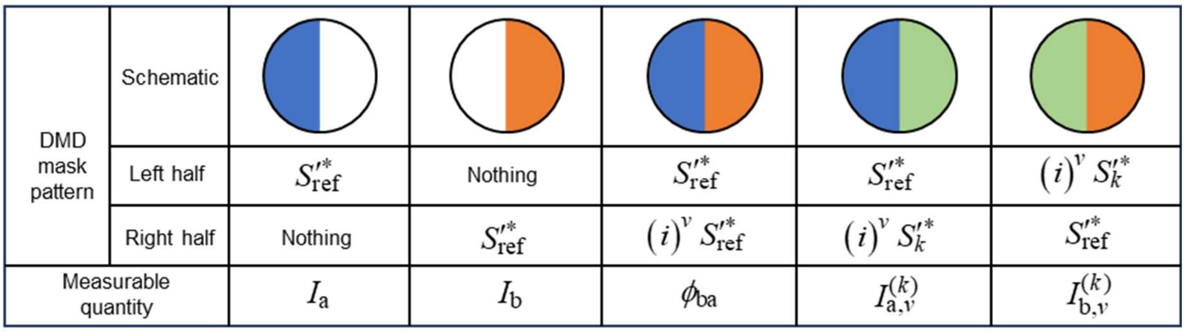				
	Left half	S'^*_{ref}	Nothing	S'^*_{ref}	S'^*_{ref}	$(i)^{\nu}\ S'^*_k$
	Right half	Nothing	S'^*_{ref}	$(i)^{\nu}\ S'^*_{\mathrm{ref}}$	$(i)^{\nu}\ S'^*_k$	S'^*_{ref}
Measurable quantity		I_{a}	I_{b}	ϕ_{ba}	$I^{(k)}_{\mathrm{a},\nu}$	$I^{(k)}_{\mathrm{b},\nu}$

Figure S13. Mask design for 2D limited-size object microscopy (LSOM). (a) Indexing of Slepian–Pollak modes k, in correspondence with main text Ref. 30. (b) Complex-conjugated and normalized (maximum modulus = 1) Slepian–Pollak functions S'^*_k used in the masks. (c) Realized mask patterns and the corresponding measurable quantities for each mask.

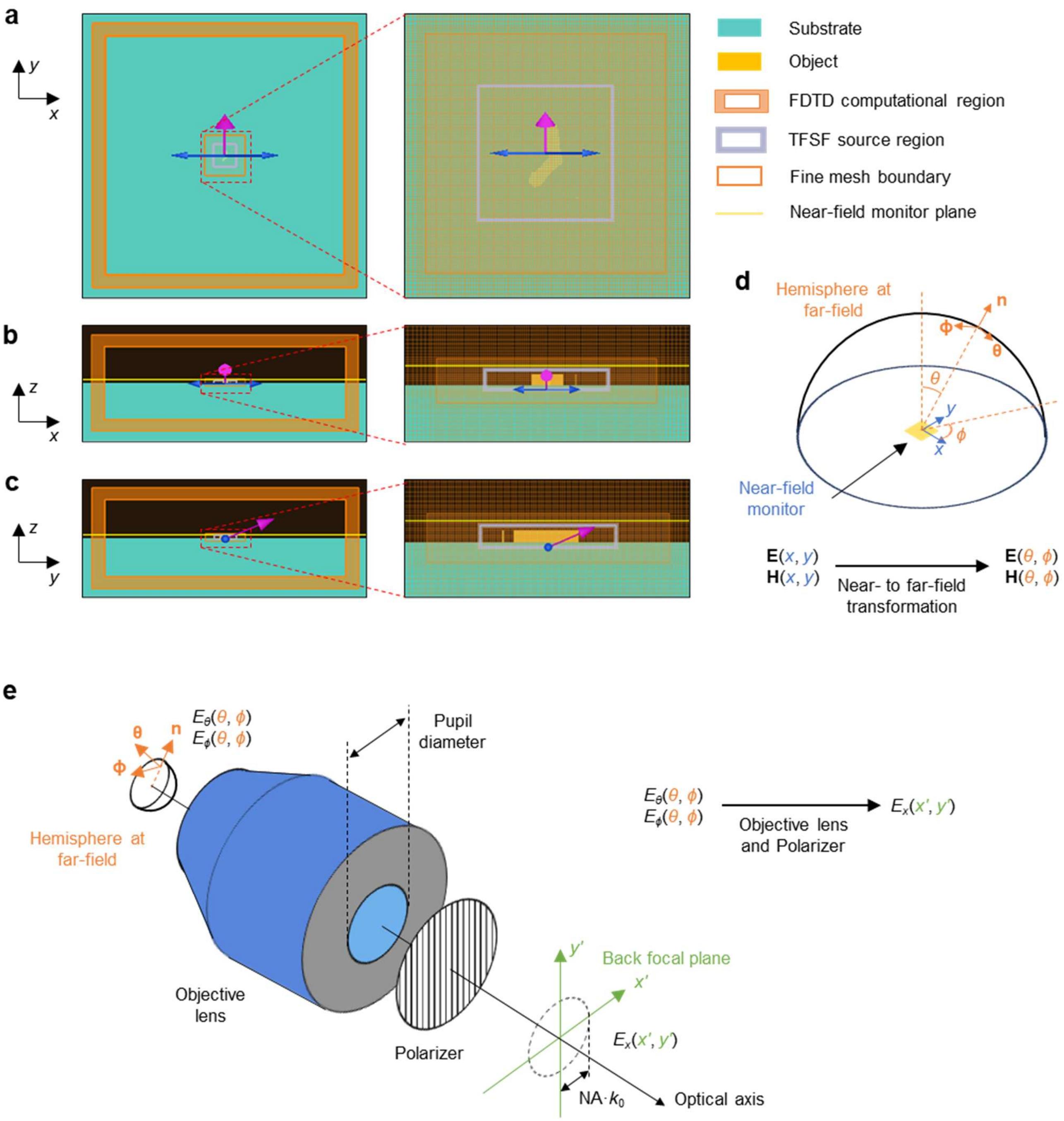

Figure S14. Finite-difference time-domain (FDTD) simulation geometry and Fourier-optic calculation. (a–c) FDTD simulation domain (to scale): 6 μm × 6 μm × 2 μm region containing the object, substrate and total-field scattered-field (TFSF) source. (d) Near-field data are transformed into the far field using the built-in near-to-far-field transform. (e) Assuming an ideal objective lens and polarizer, the E_x component in the back focal plane is computed and used as the input to the Fourier-optic calculations.

Supplementary References

1. Shechtman, Y., Sahl, S. J., Backer, A. S. & Moerner, W. E. Optimal Point Spread Function Design for 3D Imaging. *Phys. Rev. Lett.* **113**, 133902 (2014).
2. Barton, G. *Elements of Green's functions and propagation: potentials, diffusion, and waves*. (Oxford University Press, 1989).
3. Born, M. & Wolf, E. *Principles of optics: electromagnetic theory of propagation, interference and diffraction of light*. (Elsevier, 2013).
4. Potton, R. J. Reciprocity in optics. *Rep. Prog. Phys.* **67**, 717–754 (2004).
5. Helstrom, C. W. Quantum detection and estimation theory. *J. Stat. Phys.* **1**, 231–252 (1969).
6. Braunstein, S. L., Caves, C. M. & Milburn, G. J. Generalized Uncertainty Relations: Theory, Examples, and Lorentz Invariance. *Ann. Phys.* **247**, 135–173 (1996).
7. Kay, S. M. *Fundamentals of Statistical Signal Processing: Estimation Theory*. (Prentice Hall, 1993).
8. Gagliardi, R. M. & Karp, S. *Optical communications*. (Wiley, 1976).
9. Boyd, J. P. Prolate spheroidal wavefunctions as an alternative to Chebyshev and Legendre polynomials for spectral element and pseudospectral algorithms. *J. Comput. Phys.* **199**, 688–716 (2004).
